# Synergistic Effect of β-Lapachone and Aminooxyacetic Acid on Central Metabolism in Breast Cancer

**DOI:** 10.3390/nu14153020

**Published:** 2022-07-22

**Authors:** Mario C. Chang, Rohit Mahar, Marc A. McLeod, Anthony G. Giacalone, Xiumei Huang, David A. Boothman, Matthew E. Merritt

**Affiliations:** 1 Department of Biochemistry and Molecular Biology, College of Medicine, University of Florida, Gainesville, FL 32610, USA; marioc14@ufl.edu (M.C.C.); rmahar@ufl.edu (R.M.); marc.mcleod@ufl.edu (M.A.M.); anthonygiacalone@ufl.edu (A.G.G.); 2 Department of Radiation Oncology, Melvin and Bren Simon Comprehensive Cancer Center, Indiana University School of Medicine, Indianapolis, IN 46202, USA; xiuhuang@iu.edu

**Keywords:** biogenic, synergy, cancer, metabolism, GC-MS, isotopologue

## Abstract

The compound β-lapachone, a naturally derived naphthoquinone, has been utilized as a potent medicinal nutrient to improve health. Over the last twelve years, numerous reports have demonstrated distinct associations of β-lapachone and NAD(P)H: quinone oxidoreductase 1 (NQO1) protein in the amelioration of various diseases. Comprehensive research of NQO1 bioactivity has clearly confirmed the tumoricidal effects of β-lapachone action through NAD^+^-keresis, in which severe DNA damage from reactive oxygen species (ROS) production triggers a poly-ADP-ribose polymerase-I (PARP1) hyperactivation cascade, culminating in NAD^+^/ATP depletion. Here, we report a novel combination strategy with aminooxyacetic acid (AOA), an aspartate aminotransferase inhibitor that blocks the malate-aspartate shuttle (MAS) and synergistically enhances the efficacy of β-lapachone metabolic perturbation in NQO1^+^ breast cancer. We evaluated metabolic turnover in MDA-MB-231 *NQO1*^+^, MDA-MB-231 *NQO1*^−^, MDA-MB-468, and T47D cancer cells by measuring the isotopic labeling of metabolites from a [U-^13^C]glucose tracer. We show that β-lapachone treatment significantly hampers lactate secretion by ~85% in NQO1^+^ cells. Our data demonstrate that combinatorial treatment decreases citrate, glutamate, and succinate enrichment by ~14%, ~50%, and ~65%, respectively. Differences in citrate, glutamate, and succinate fractional enrichments indicate synergistic effects on central metabolism based on the coefficient of drug interaction. Metabolic modeling suggests that increased glutamine anaplerosis is protective in the case of MAS inhibition.

## 1. Introduction

β-lapachone (β-lap) is a biogenic naphthoquinone naturally produced in the bark of the lapacho tree (*Tabebuia avellanedae*) [1]. The extract of this plant has been used for thousands of years as a curative medicine and as a nutrient for improving health [2]. Indigenous people of South and Central American countries have used the bark to remedy several health conditions through inherent antibacterial, antifungal, anti-inflammatory, analgesic, and anti-nociceptive effects; all of which have been confirmed by modern-day research [3,4,5,6]. Between the last decade and recent years, several in vitro and in vivo studies have reported associations of β-lap treatment with NAD(P)H: quinone oxidoreductase 1 (NQO1) expression in the attenuation of various diseases and health conditions [7]. NQO1 is a two-electron reductase globally expressed in most eukaryotes at relatively low levels to protect against oxidative stress by detoxifying the cytoplasmic environment [8]. β-lap associated activity at NQO1 has been shown to modulate NAD^+^/NADH metabolism resulting in the prevention of cognitive and motor deterioration [7,9], protection against nephrotoxicity induced by acute kidney injury [10,11], reduction in inflammation through the immunomodulation of arthritis [12], and promotion of wound healing [13]. β-lap is thus highlighted as a supplement for overall better health.

While numerous studies have reported the beneficial effects of β-lap treatment against a wide variety of ailments, extensive research of NQO1 bioactivation has revealed strong anti-cancer effects of β-lap as well [14,15,16,17,18]. Chemotherapeutic treatment of cancers with β-lap bioactivates NQO1, yielding a highly unstable hydroquinone that auto-oxidizes in an aggressive futile cycle and generates large amounts of reactive oxygen species (ROS) that are ultimately transformed to hydrogen peroxide [19,20,21]. The accelerated accretion of hydrogen peroxide causes overwhelming DNA damage, which hyperactivates poly-ADP-ribose polymerase-I (PARP1), inducing substantial global depletion of NAD^+^ and ATP, thus hindering glycolysis, cellular redox balance, and downstream metabolism [22,23]. Through this mechanism, cancer cells that overexpress NQO1 consequently die from NAD^+^-keresis [24]. The significant anti-proliferative effects on cancer cells induced by β-lap treatment have been shown in a variety of NQO1^+^ cancers including, lung, pancreatic, head and neck, prostate, colon, and breast cancers [25].

Although β-lap has shown compelling promise against carcinogenesis and tumorigenesis in murine models, and in Phase-1 clinical trials, it has demonstrated serious dose-dependent off-target toxicity in red blood cells in the form of methemoglobinemia and hemolytic anemia, bringing about serious concerns for drug safety [26,27]. Therefore, identifying ways of reducing drug toxicity while maximizing therapeutic effects would prove highly beneficial for overall patient outcome under a β-lap therapeutic regimen. One way of achieving this would be through the identification of compounds synergistic with β-lap. Previous studies have identified synergistic combinations with β-lap by targeting DNA repair and metabolic vulnerabilities via X-ray repair cross complementing 1 (XRCC1) [14], PARP1 [25], proliferating cell nuclear antigen (PCNA) [28], and glutamine metabolism [29], all associated with aspects of the NAD^+^-keresis mechanism.

Here, we report a novel combination with aminooxyacetic acid (AOA), a malate-aspartate shuttle (MAS) inhibitor, which enhances β-lap specific metabolic downregulation in NQO1^+^ breast cancer. In theory, augmenting β-lap treatment with AOA would further drive the perturbation of the cellular NAD^+^/NADH redox balance, as the MAS regulates NAD^+^/NADH homeostasis between the mitochondrial and cytosolic compartments [30].

To this end, we utilized MDA-MB-231 *NQO1*^+^, MDA-MB-231 *NQO1*^−^, MDA-MB-468, and T47D breast cancer cells to test the hypothesis that β-lap and AOA combinatorial treatment synergistically reduces metabolic turnover in an NQO1 dependent manner. We targeted an MDA-MB-231 breast cancer cell model because it has already been shown to be sensitive to the AOA-dependent inhibition of aspartate aminotransferase (AST), a principal component of the MAS [31]. MDA-MB-231 *NQO1*^+^, triple-negative breast cancer cells express the highest level of NQO1 protein and activity, while MDA-MB-231 *NQO1*^−^ are isogenic knockouts with zero NQO1 activity [32]. MDA-MB-468 triple-negative breast cancer cells are deficient for NQO1, whereas T47D ER and PR positive luminal breast cancer cells express low levels of NQO1 with diminished activity [8]. Experimentally, we assessed metabolic health by quantitatively measuring isotopologue labeling of central metabolites generated from a [U-^13^C]glucose tracer. The fractional enrichment of citrate, glutamate, and succinate isotopologues demonstrated a synergistic downregulation of central metabolism as determined through the calculation of the coefficient of drug interactions. We have previously shown a significant β-lap-dependent reduction in isotopic labeling in citrate and other central metabolites utilizing a [^2^H_7_]glucose tracer [23]. Here, we enhance the metabolic effects of β-lap by combining treatment with a sublethal dose of AOA and demonstrate a proof of principle for a plausible and novel combination strategy targeting breast cancer metabolic vulnerability.

## 2. Materials and Methods

### 2.1. Cell Lines and Reagents

MDA-MB-231 *NQO1*^+^ and MDA-MB-231 *NQO1*^−^ isogenic triple-negative breast cancer cell lines were generously gifted by the late Dr. David Boothman. T47D, estrogen receptor (ER) and progesterone receptor (PR) positive, luminal breast cancer cells were graciously donated by Dr. Susan Frost. MDA-MB-468 triple-negative breast cancer cells were purchased from the American Type Culture Collection (ATCC, Manassas, VA, USA). Roswell Park Memorial Institute Medium (RPMI 1640), Phosphate Buffered Saline (PBS), HEPES (N-2-hydroxyethylpiperazine-N-2-ethane sulfonic acid), Penicillin-Streptomycin-Neomycin (PSN), Trypsin-Ethylenediaminetetraacetic acid (EDTA), and Dimethyl Sulfoxide (DMSO) were acquired from Thermo Scientific (Waltham, MA, USA). Sterile Filtered Fetal Bovine Serum (FBS) was commercially attained from Atlas Biological (Fort Collins, CO, USA). [U-^13^C]glucose was purchased from Cambridge Isotope Laboratories (Tewksbury, MA, USA). β-lapachone (2,2-dimethyl-3,4-dihydrobenzo[h]chromene-5,6-dione) was obtained from Sigma Aldrich (St. Louis, MO, USA). Aminooxyacetic Acid (2-(aminooxy)-acetic acid) was purchased from Cayman Chemical (Ann Arbor, MI, USA). Methoxyamine hydrochloride dissolved in pyridine (MOX) and N-methyl-N-(tert-butyldimethylsilyl)-trifluoroacetamide + 1% tertbutyldimethylchlorosilane (MTBSTFA + TBDMS) were commercially attained from Thermo Scientific (Waltham, MA, USA).

### 2.2. Cell Culture Experiment

All cell lines were cultured in complete growth media consisting of high glucose Roswell Park Memorial Institute Medium (RPMI 1640) with 10% FBS (*v*/*v*), 10 mM HEPES, 50 µg/mL streptomycin, 50 µg/mL penicillin, and 10 µg/mL neomycin. Cells were maintained at 37 °C and 5% carbon dioxide (CO_2_) in a Heracell Vios 160i air-jacketed incubator (Thermo Scientific, Waltham, MA, USA). Prior to experiments, complete growth media was replaced every 2–3 days. At 80% confluency, cells were passaged 1:10 into twelve 100 mm diameter (58.1 cm^2^ culture area) tissue culture dishes. All cell lines were cultured to 70% confluency (~8 million cells), washed with warm PBS, and incubated for 2 h with 10 mL of complete RPMI with 10 mM [U-^13^C]glucose and the following treatments: DMSO and PBS vehicle control, 6 µM β-lap in DMSO, 100 µM AOA in PBS, or a combination of 6 µM β-lap and 100 µM AOA. The concentration of β-lap (6 µM) and AOA (100 µM) were chosen to impart a significant metabolic insult without decrements in cell death [22], and to target AST inhibition [31], respectively. After the 2 h treatment, media was removed, cells were washed with warm PBS, and 10 mL of untreated RPMI 1640 with 10 mM [U-^13^C]glucose were added. Cell lines were incubated in untreated media with a tracer for 2 h. During the 2 h incubation, 200 μL aliquots of media were sampled at 0 min, 15 min, 60 min, and 120 min (2 h) and stored a −80 °C for further analysis. At the end of the 2 h incubation, all media was collected, cells were washed with warm PBS, and harvested by trypsinization (Supplementary Materials Figure S1). Cells were then pelleted by centrifugation at 4 °C at 200× *g* for 3 min, supernatant was aspirated, and pellets were washed 2× with 0.9% saline and flash frozen in liquid nitrogen (N_2_) for subsequent gas chromatography–mass spectrometry (GC-MS) analysis.

### 2.3. Extraction of Metabolites and Sample Preparation for GC-MS Analysis

Media samples were thawed at room temperature, briefly vortexed, and 20 µL were transferred and dried in Reacti-Vial^TM^ reaction vials (Thermo Scientific, Waltham, MA, USA) with N_2_ air flow. Cell pellets were thawed on ice and extracted with 1.5 mL of Acetonitrile:Isopropanol:Water (3:3:2, *v*/*v*/*v*) with D,L-Norleucine internal standard. Cell debris was pelleted from cell extracts by centrifugation at 10,000× *g* at 4 °C for 25 min. Supernatants were collected and pipetted into a new microcentrifuge tube and dried in a speedvac. Dried cell extracts were reconstituted in 0.4 mL of Acetonitrile:Water (1:1, *v*/*v*). Reconstituted extracts were centrifuged at 10,000× *g* at 4 °C for 25 min and supernatants were transferred to Reacti-Vial^TM^ reaction vials (Thermo Scientific, Waltham, MA, USA) and dried with N_2_ air flow. Once dried, 50 µL of 2% MOX derivatizing reagent was added to each sample vial, vortexed for 5 s, and incubated at 30 °C for 2 h on a Reacti-Therm III^TM^ (Thermo Scientific, Waltham, MA, USA) heating module with a magnetic stir bar for even mixing of sample reactions. At the end of the 2 h incubation, samples were derivatized with 50 µL of MTBSTFA + TBDMS for 1 h incubation at 60 °C. Sample reaction vials were then centrifuged at 8000× *g* for 10 min and supernatants were transferred into GC-MS sample vials.

### 2.4. GC-MS Analysis

GC-MS analysis was conducted on a Thermo Scientific ISQ LT Single Quadrupole Mass Spectrometer and Trace 1310 Gas Chromatograph (GC-MS, Thermo Scientific, Waltham, MA, USA) equipped with 30 m long Restek 95% dimethyl: 5% diphenyl polysiloxane RTX-5MS column, with a 0.25 mm internal diameter, 0.25 µm film, and a 10 m guard column (Restek, Bellefonte, PA, USA). Chromatographic separation was achieved with the following parameters: initial oven temperature of GC was 60 °C for 60 s followed by an iterative increase (10 °C/min) in the ramp oven temperature to reach 325 °C with a 5 min final hold time in the end. Subsequently, metabolites were ionized with an electron impact (EI) ion source maintained at 230 °C with an applied electron ionization energy of 70 eV. MS separation of ions was performed with a single quadrupole mass analyzer and helium gas was utilized as a carrier with a flow rate of 1 mL/min.

### 2.5. Data Processing, Isotopologue Analysis, and Flux Modeling Analysis

Mass spectrometry data were recorded using Xcalibur 4.5 software (Thermo Scientific, Waltham, MA, USA). Chromatograms were analyzed with Qual Browser and metabolite peaks were identified using the National Institute of Standards and Technology (NIST) library [33]. Extracted ion chromatograms were integrated into Quan Browser to obtain the Mass Isotopomer Distribution (MID) of each identified metabolite. The MID of each metabolite was corrected for natural isotope abundance with the Isotopomer Network Compartmental Analysis software (INCA 2.0, Vanderbilt University, Nashville, TN, USA) [34,35]. INCA was then utilized to assemble an isotopomer network model to estimate the relative net flux values of central metabolic reactions outlined in Supplementary Materials Figure S7 and Table S2. Extracellular and intracellular isotopologue data, as well as relative net flux data, were plotted and assessed by 2-way ANOVA and student’s *t*-test post hoc analysis using GraphPad Prism 6 software (GraphPad, La Jolla, CA, USA).

### 2.6. Multivariate Statistical Analysis

Multivariate statistical analysis was applied to the GC-MS data sets of all cell extract samples. The intensity of each of the intracellular metabolites was normalized to the intensity of D,L-Norleucine. MetaboAnalyst 5.0 (Xia Lab, McGill University, Ste. Anne de Bellevue, Montreal, QC, Canada, www.metaboanalyst.ca (accessed on 1 October 2021)) [36] was utilized to perform principal component analysis (PCA) on GC-MS data after processing by total sum normalization, log10 transformation, and pareto scaling. 

### 2.7. Coefficient of Drug Interaction

Coefficient of drug interaction (CDI) values was calculated to assess the synergistic effects of combinatorial treatment. CDI values were calculated with the following formula: CDI = AB/(A × B). In this formula, AB is the ratio of β-lap+AOA to control, A is the ratio of AOA to control, and B is the ratio of β-lap to control for the fractional enrichment of metabolites. In this calculation, a value of CDI < 1 indicates synergy, CDI = 1 indicates an additive effect, and CDI > 1 indicates an antagonistic effect.

### 2.8. NQO1 Protein Expression Analysis

All four cell lines were screened for NQO1 protein expression levels by Western-blot analysis. The cell lines were harvested at 80% confluency and lysed in cold RIPA buffer containing Halt protease and phosphatase inhibitor (Thermo Scientific, Waltham, MA, USA). A Bradford assay (Bio-Rad, Hercules, CA, USA) was performed to determine the protein concentration of each lysate for loading normalization. Normalized lysates were loaded in a 4–15% Criterion TGX gel (Bio-Rad, Hercules, CA, USA) and protein separation was achieved by running gels at a constant 0.05 A. Gels were then transferred to a polyvinylidene fluoride (PVDF) membrane (Bio-Rad, Hercules, CA, USA) at 50 V at 4 °C. The membrane was washed and blocked with 5% non-fat dry milk dissolved in TBS-T (200 mM sodium chloride (NaCl), 30 mM Tris-hydrochloric acid (HCl) (pH 7.6), and 0.1% Tween-20) for 1 h. Proteins were probed with primary NQO1 and α-tubulin mouse monoclonal antibodies (Cell Signaling Technology, Danvers, MA, USA). Primary antibodies were diluted (NQO1 1:1000 *v*/*v* and α-tubulin 1:5000 *v*/*v*) with 5% non-fat dry milk dissolved in TBS-T and separately incubated with the blot for 18 h at 4 °C. After each primary antibody incubation, the blot was washed and then incubated in secondary anti-mouse horseradish peroxidase (HRP) antibody (diluted 1:5000 *v*/*v*) at room temperature for 1 h. At the end of the incubation, the membrane was washed and detected with Pierce^TM^ ECL Western Blotting Substrate (Thermo Scientific, Waltham, MA, USA) followed by exposure to PRIMA1 autoradiography film (Midwest Scientific, Valley Park, MO, USA).

### 2.9. Cell Viability Assay

An MTT assay was performed to assess the effects of β-lap and AOA on cell viability. Cells were subcultured into clear 96 well plates and incubated overnight. Cells were then pre-treated with complete media containing either a vehicle control or 100 μM AOA for 2 h. Then the cells were washed with PBS and treated with a vehicle control of 6 μM β-lap for 2 h. At the end of the incubation, treatment media was removed and fresh media with MTT was added for 3 h. The media was then removed and 100% DMSO was added to each sample well. The plates were incubated at room temperature and shaken for 30 min. Absorbance at 570 nm was then measured using a Synergy 2 plate reader (BioTek, Winooski, VT, USA).

## 3. Results

### 3.1. Global Metabolomics

**Figure 1:**
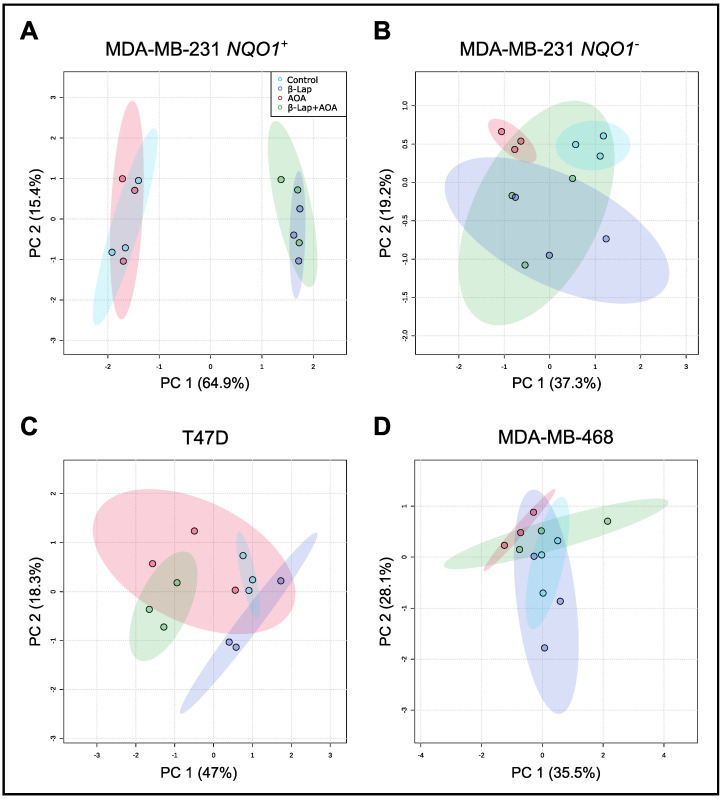
PCA analysis of GC-MS global metabolic profiles of four different breast cancer cell lines treated with either 6 µM β-lap, 100 µM AOA, or a combination of both drugs. Metabolites were analyzed based on internal standard normalized intensity. Panel (**A**) shows clear separation in MDA-MB-231 *NQO1*^+^ cells driven by β-lap treatment. Panel (**B**) shows much-reduced clustering in β-lap and β-lap+AOA treatment due to lack of NQO1 in MDA-MB-231 *NQO1*^−^ cells. Panel (**C**) shows some separation among control, β-lap, and β-lap+AOA groups in T47D cells. Panel (**D**) shows limited effects in MDA-MB-468 cells, which have almost no NQO1 expression. PCA, principal component analysis; GC-MS, gas chromatography–mass spectrometry; β-lap, β-lapachone; AOA, aminooxyacetic acid; NQO1, quinone oxidoreductase 1.

Multivariate statistical analysis was applied to the GC-MS data set to produce a global overview of the metabolic profiles of cancer cells under control and treatment conditions. A principal component analysis (PCA) scores plot separated treatment groups based on β-lap sensitivity, corresponding to NQO1 expression and activity (Figure 1). MDA-MB-231 *NQO1*^+^ cells showed the greatest separation along PC 1 (64.9%) driven by β-lap drug action as evidenced by relative clustering of Control/AOA and β-lap/β-lap+AOA treatment groups, respectively (Figure 1A). In contrast, treatment groups were clustered in MDA-MB-231 *NQO1*^−^ cells due to the absence of β-lap drug action (Figure 1B). Distinct separation among control, β-lap, and β-lap+AOA groups was observed in T47D cells (Figure 1C), while AOA treatment showed higher variation, overlapping with all groups. MDA-MB-468 cells showed overlap among all groups, consistent with the low expression of NQO1 (Figure 1D).

### 3.2. Fractional Enrichment of ^13^C-lactate and ^13^C-alanine Isotopologues

**Figure 2:**
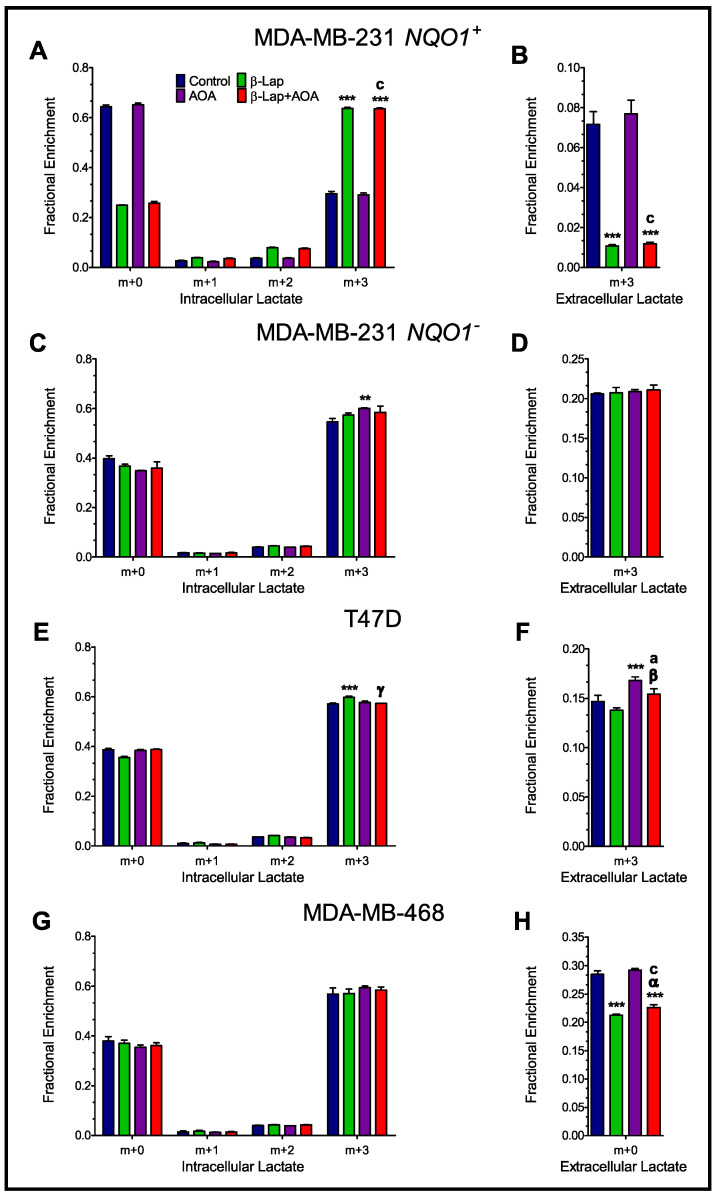
Evaluating glycolytic output by analyzing the intracellular and extracellular isotopologues of lactate in MDA-MB-231 *NQO1*^+^ (Panels (**A**,**B**)) and MDA-MB-231 *NQO1*^−^ cells (Panels (**C**,**D**)). β-lap action drives glycolytic slowdown in NQO1^+^ cells as evidenced by significantly lower lactate secretion supported by higher intracellular enrichment of m+3 lactate in β-lap and combined treatment compared to control, and significantly lower extracellular enrichment of m+3 lactate in the same groups. Conversely, treatment was inconsequential across groups in NQO1^−^ cells. Intracellular and extracellular lactate isotopologue distribution in T47D are shown in (Panels (**E**,**F**)) and MDA-MB-468 in (Panels (**G**,**H**)) breast cancer cells. (Note: *n* = 3, biological replicate data are represented as mean ± SEM. Statistical significance was determined by ANOVA and student’s *t*-test post hoc analysis and is presented as: (**) if *p ≤* 0.01 and (***) if *p ≤* 0.001 compared to control. (α) if *p ≤* 0.05, (β) if *p ≤* 0.01, and (γ) if *p ≤* 0.001 compared to β-lap treatment. (a) if *p ≤* 0.05 and (c) if *p ≤* 0.001 compared to AOA treatment). SEM, standard error of the mean.

To assess glycolytic turnover, the fractional enrichment of ^13^C-lactate and ^13^C-alanine isotopologues was evaluated for both intra- and extracellular compartments. In Figure 2, panels A and B show that β-lap powerfully modifies glucose handling in MDA-MB-231 *NQO1*^+^ cells, as evidenced by significantly higher intracellular enrichment of m+3 lactate in β-lap and combined treatment compared to control, as well as significantly lower extracellular enrichment of m+3 lactate in β-lap and combined treatment compared to control, matching the trend observed in normalized lactate pool concentration (Supplementary Materials Figure S2). In contrast, drug action was negligible in the case of MDA-MB-231 *NQO1*^−^ cells (Figure 2C,D). T47D cells under combinatorial treatment showed a significant decrease in intracellular m+3 lactate fractional enrichment compared to β-lap and a significant increase in extracellular m+3 lactate fractional enrichment compared to AOA (Figure 2E,F). In contrast, MDA-MB-468 cells showed no significant differences across all three treatment groups in intracellular lactate isotopologue distribution and significantly lower enrichment of extracellular m+3 lactate in β-lap and β-lap+AOA groups compared to control (Figure 2G,H).

**Figure 3:**
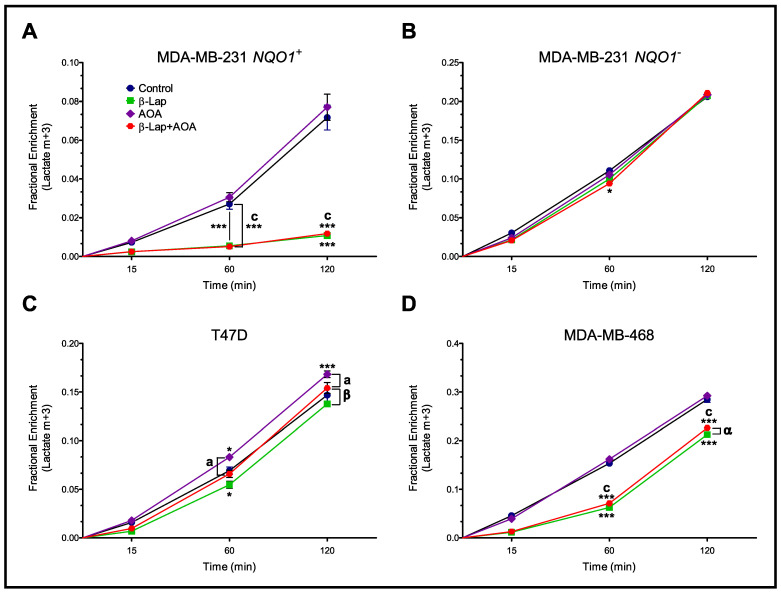
Kinetic analysis of extracellular lactate m+3 labeling over the course of 2 h after treatment. Panel (**A**) shows highly differential lactate production rates for MDA-MB-231 *NQO1*^+^ cells. In panel (**B**), MDA-MB-231 *NQO1*^−^ showed no drastic differences across treatment groups. T47D showed slight differences among treatment groups (Panel (**C**)). MDA-MB-468 cells demonstrated lower production rates for β-lap and β-lap+AOA treatments (Panel (**D**)). (Note: *n* = 3, biological replicate data are represented as mean ± SEM. Statistical significance was determined by ANOVA and student’s *t*-test post hoc analysis and is presented as: (*) if *p ≤* 0.05 and (***) if *p ≤* 0.001 compared to control. (α) if *p ≤* 0.05 and (β) if *p ≤* 0.01 compared to β-lap treatment. (a) if *p ≤* 0.05 and (c) if *p ≤* 0.001 compared to AOA treatment).

To further address glycolytic perturbations, a kinetic analysis of extracellular lactate production was performed (Figure 3). GC-MS analysis was performed on cell media samples at 15, 60, and 120 min time points (post-treatment), and lactate m+3 fractional enrichments were plotted over that time. MDA-MB-231 *NQO1*^+^ cells showed the greatest differences in lactate production between Control/AOA and β-lap/β-lap+AOA treatment groups (Figure 3A), indicating that β-lap dominated reductions in lactate enrichment. On the other hand, MDA-MB-231 *NQO1*^−^ cells showed essentially no difference in lactate enrichment rate as evidenced by linear plots with almost identical slopes across all three treatment groups (Figure 3B). T47D cells showed a similar trend with the lowest lactate enrichment rate observed for the β-lap-treated group (Figure 3C). MDA-MB-468 showed slightly lower lactate m+3 enrichment generated over time in β-lap and β-lap+AOA treatment groups (Figure 3D).

**Figure 4:**
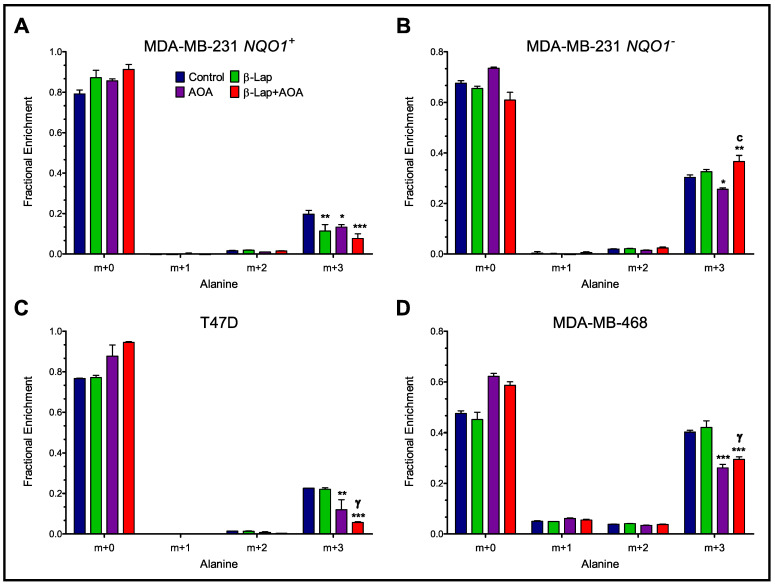
Intracellular alanine isotopologue distribution in MDA-MB-231 *NQO1*^+^ (Panel (**A**)), MDA-MB-231 *NQO1*^−^ (Panel (**B**)), T47D (Panel (**C**)), and MDA-MB-468 (Panel (**D**)). Alanine MID reflects treatment effects on a branching pathway of glycolysis. (Note: *n* = 3, biological replicate data are represented as mean ± SEM. Statistical significance was determined by ANOVA and student’s *t*-test post hoc analysis and is presented as: (*) if *p ≤* 0.05, (**) if *p ≤* 0.01, and (***) if *p ≤* 0.001 compared to control. (γ) if *p ≤* 0.001 compared to β-lap treatment. (c) if *p ≤* 0.001 compared to AOA treatment).

As AOA is expected to inhibit transamination reactions, alanine fractional enrichment was analyzed to differentiate effects from interrupted glycolysis versus nitrogen metabolism. Analysis of alanine isotopologue labeling revealed AOA-dependent metabolic effects. All four cell lines treated with AOA showed significantly lower intracellular enrichment of m+3 alanine labeling compared to control (Figure 4). As expected, MDA-MB-231 *NQO1*^+^ cells showed significantly lower m+3 labeling in β-lap treatment and the most significant decrease in combinatorial treatment compared to control (Figure 4A). MDA-MB-231 *NQO1*^−^ cells showed no significant difference between control and β-lap, but instead significantly higher m+3 labeling in combinatorial treatment compared to control and AOA treatments, respectively (Figure 4B). T47D showed a synergistic effect (CDI = 0.49 ± 0.02) on m+3 alanine labeling, which reflects significantly lower labeling in combinatorial treatment compared to control and β-lap groups (Figure 4C). MDA-MB-468 cells showed the most significant decrease in m+3 alanine labeling in AOA compared to control (Figure 4D). The labeling data for all four cell lines matched the trends observed in the normalized alanine pools (Supplementary Materials Figure S2).

### 3.3. Fractional Enrichment of TCA Cycle Intermediates

**Figure 5:**
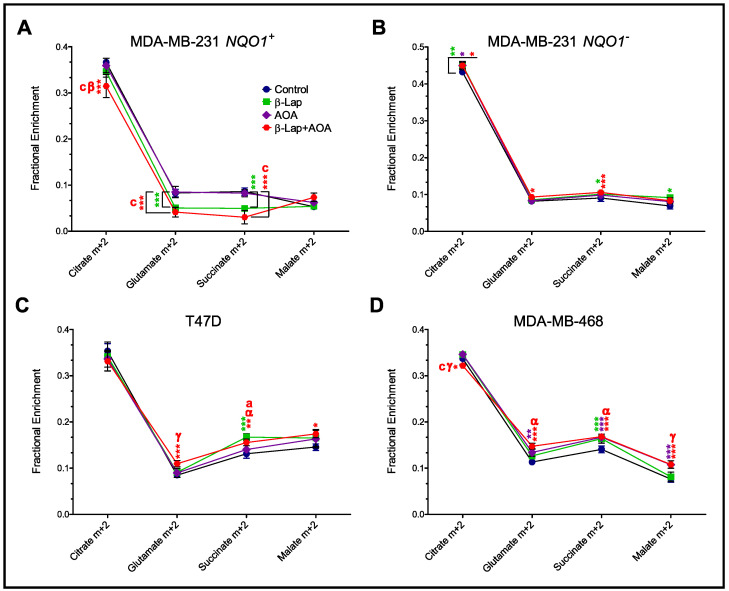
Assessment of TCA cycle activity by measuring isotopologue labeling in TCA cycle metabolites revealed differences in the fractional enrichment of the m+2 isotopologues of citrate, glutamate, succinate, and malate in MDA-MB-231 *NQO1*^+^ (Panel (**A**)), MDA-MB-231 *NQO1*^−^ (Panel (**B**)), T47D (Panel (**C**)), and MDA-MB-468 (Panel (**D**)). Results were calculated from intracellular GC-MS data and corrected for natural isotope abundance using the Isotopomer Network Compartmental Analysis (INCA 2.0) software. (Note: *n* = 3, biological replicate data are represented as mean ± SD. Statistical significance was determined by ANOVA and student’s t-test post hoc analysis and is presented as: (*) if *p ≤* 0.05, (**) if *p ≤* 0.01, and (***) if *p ≤* 0.001 compared to control. (α) if *p ≤* 0.05, (β) if *p ≤* 0.01, and (γ) if *p ≤* 0.001 compared to β-lap treatment. (a) if *p ≤* 0.05 and (c) if *p ≤* 0.001 compared to AOA treatment. Significance marks were color-coded by treatment). TCA, tricarboxylic acid; SD, standard deviation.

Mass isotopologue analysis revealed distinct treatment-dependent differences in the fractional enrichment of the m+2 isotopologues of citrate, glutamate, succinate, and malate in MDA-MB-231 *NQO1*^+^ cells (Figure 5). Results were calculated from the GC-MS data of cell samples harvested at the end of the experiment (2 h post-treatment incubation). Lower enrichment of citrate, glutamate, and succinate m+2 isotopologues was found in the combined treatment group compared to all other treatments in MDA-MB-231 *NQO1*^+^ cells (Figure 5A) indicating a potential synergistic effect on central metabolism. Furthermore, approximately equal enrichment of all m+2 isotopologues for all three treatment groups was found in MDA-MB-231 *NQO1*^−^ cells, suggesting minor to no differences in tricarboxylic acid (TCA) cycle metabolism in the absence of NQO1 activity (Figure 5B). The differential enrichment of m+2 isotopologues was observed across all treatment groups, likely caused by intrinsic metabolic differences in T47D cells (Figure 5C), whereas minor differences in the labeling of TCA cycle metabolites were observed in MDA-MB-468 cells across all treatment groups (Figure 5D). An assessment of aspartate labeling in all four cell lines showed clear differences among all three treatment groups (Supplementary Materials Figure S3). Both MDA-MB-231 *NQO1*^+^ and *NQO1*^−^ cell lines showed increases in aspartate m+2 labeling in a β-lap-dependent manner (Supplementary Materials Figure S3A,B). On the other hand, T47D and MDA-MB-468 cells showed non-significant differences across all three treatments, with MDA-MB-468 showing an increasing labeling trend in an AOA-dependent manner (Supplementary Materials Figure S3C,D).

**Figure 6:**
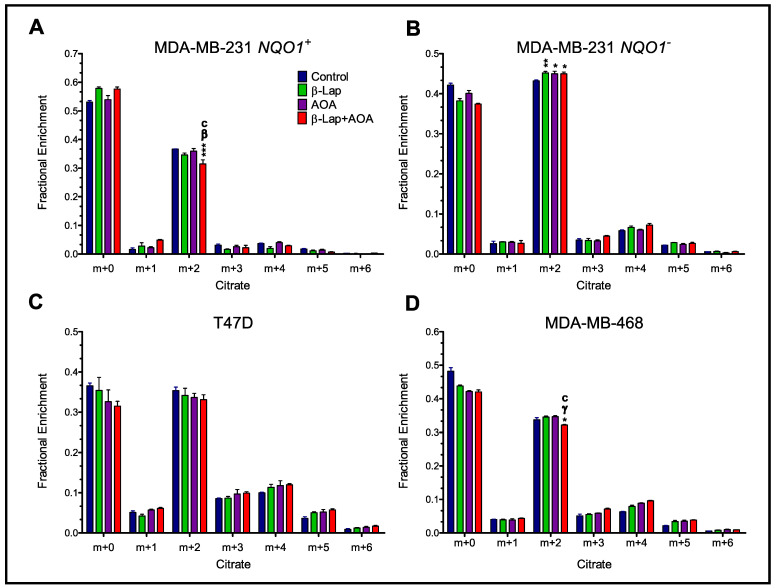
Citrate isotopologue distribution of breast cancer cells treated with β-lap and AOA. MDA-MB-231 *NQO1*^+^ (Panel (**A**)) and MDA-MB-231 *NQO1*^−^ (Panel (**B**)) cells show that AOA enhances β-lap drug action in downregulating the TCA cycle. T47D (Panel (**C**)) cells showed no significant differences in isotopologue fractional enrichments across treatments. MDA-MB-468 (Panel (**D**)) cells showed a significant decrease in citrate m+2 labeling in combinatorial treatment compared to all other groups. (Note: *n* = 3, biological replicate data are represented as mean ± SEM. Statistical significance was determined by ANOVA and student’s *t*-test post hoc analysis and is presented as: (*) if *p ≤* 0.05, (**) if *p ≤* 0.01, and (***) if *p ≤* 0.001 compared to control. (β) if *p ≤* 0.01 and (γ) if *p ≤* 0.001 compared to β-lap treatment. (c) if *p ≤* 0.001 compared to AOA treatment).

To enhance our understanding of the specific effects of combinatorial treatment against NADH-dependent pyruvate dehydrogenase (PDH) flux, we analyzed the citrate isotopologue distribution of all four cell lines (Figure 6). In MDA-MB-231 *NQO1*^+^, citrate m+2 enrichments were significantly lower for combined treatment compared to all other treatments (Figure 6A). In contrast, the drug action effects are essentially reversed in the absence of NQO1 (Figure 6B). Significant differences are observed in citrate m+2 isotopologue enrichment compared to control and AOA, likely due to off-target effects in the absence of NQO1. Citrate labeling in T47D cells (Figure 6C) showed no significant differences across treatment groups. Interestingly, MDA-MB-468 cells showed a significant decrease in citrate m+2 labeling with combinatorial treatment, but this difference was made up in the m+4 fragment, not in the m+0 peak (Figure 6D).

**Figure 7:**
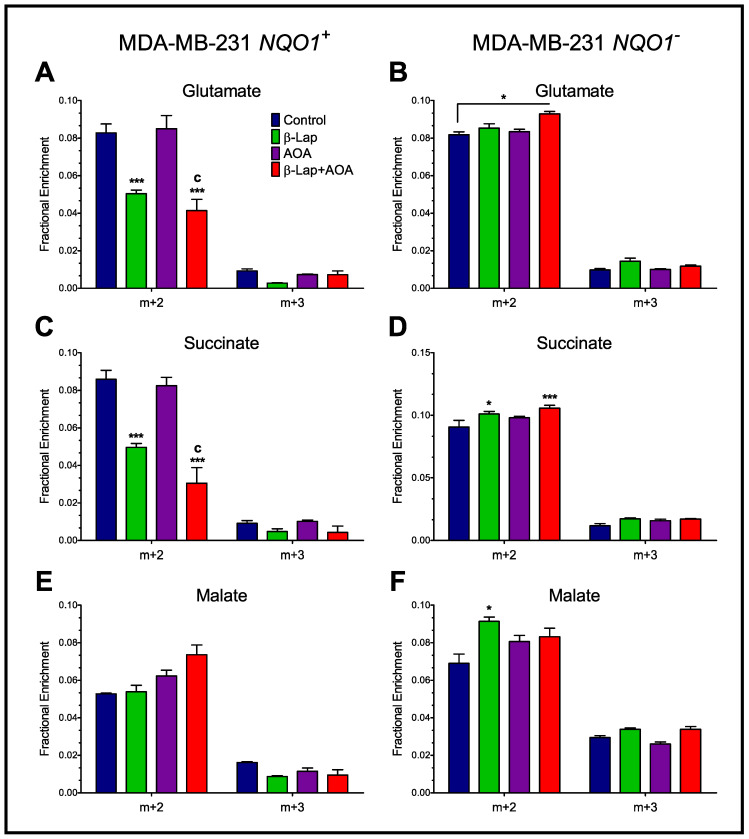
Analysis of TCA cycle activity across treatment groups by assessing the fractional enrichment of glutamate, succinate, and malate m+2 and m+3 isotopologues in MDA-MB-231 *NQO1*^+^ (Panels (**A**,**C,E**)) and MDA-MB-231 *NQO1*^−^ cells (Panels (**B**,**D,F**)). Differential labeling in intermediates demonstrates a significant reduction in TCA cycle turnover induced by β-lap and AOA combinatorial treatment. (Note: *n* = 3, biological replicate data are represented as mean ± SEM. Statistical significance was determined by ANOVA and student’s *t*-test post hoc analysis and is presented as: (*) if *p ≤* 0.05 and (***) if *p ≤* 0.001 compared to control. (c) if *p ≤* 0.001 compared to AOA treatment).

TCA cycle and transaminase activity across treatment groups was further assessed by measuring the fractional enrichment of glutamate, succinate, and malate m+2 and m+3 isotopologues in MDA-MB-231 *NQO1*^+^ and MDA-MB-231 *NQO1*^−^ cells (Figure 7). Note that, in general, fractional ^13^C enrichment was significantly lower among all metabolites downstream of citrate. In MDA-MB-231 *NQO1*^+^ cells, glutamate m+2 enrichments are significantly lower in β-lap and combined groups compared to control, indicating that β-lap is driving the metabolic perturbation. Succinate m+2 enrichments are significantly lower in the combined treatment of NQO1^+^ cells compared to control and AOA treatments, and are appreciably lower compared to β-lap treatment (Figure 7C). Malate m+2 was not significantly enriched across treatments in NQO1^+^ cells (Figure 7E). In NQO1^−^ cells, malate m+2 enrichment was significantly higher in β-lap, AOA, and combinatorial treatments compared to control (Figure 7F). This observed effect is consistent in aspartate m+2 enrichment, which is significantly higher in combinatorial treatment compared to all groups in NQO1^+^ cells (Supplementary Materials Figure S3A). Comparative analysis of m+3 enrichment of glutamate, succinate, malate, and aspartate show a very similar trend in an NQO1-dependent manner. The data consistently show that in NQO1^+^ cells, m+3 enrichment is lowest in β-lap treatment while this effect is the reverse in NQO1^−^ cells. Overall, drug action caused opposite effects in the absence of NQO1 with some significant differences across treatments in glutamate, succinate, and malate isotopologue enrichments (Figure 7 and Supplementary Materials Figure S4).

### 3.4. Metabolic Flux Analysis

**Figure 8:**
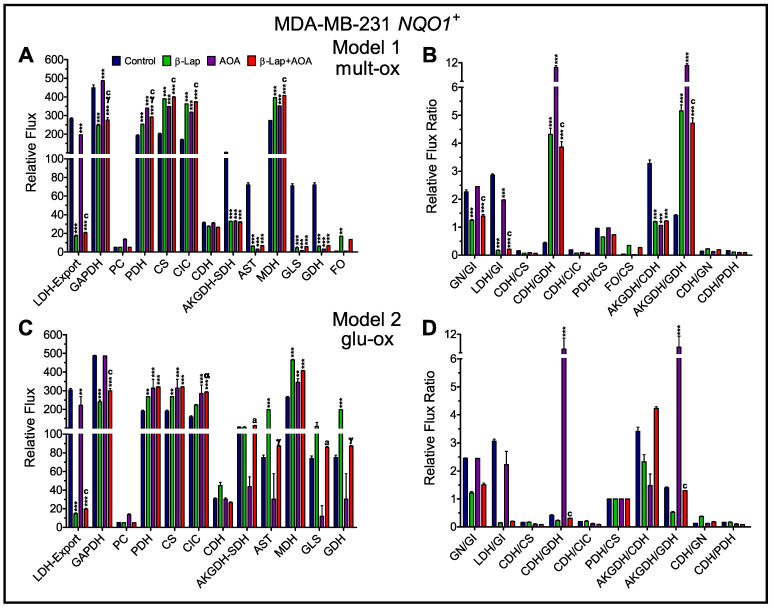
Analysis of metabolic flux in MDA-MB-231 *NQO1*^+^ cells. The relative net flux, as well as relative flux ratios, was assessed in two isotopomer network models. Model 1, mult-ox, (Panels (**A**,**B**)) addresses central metabolic pathways and reactions, as well as branching pathways relevant to the mechanism of action. Model 2, glu-ox, (Panels (**C**,**D**)) is a modified version of the first model that excludes fatty acid import and oxidation, which limits the contribution of unlabeled acetyl-CoA in the model. This alternative analysis infers greater GLS and GDH flux in β-lap-treated NQO1^+^ cells. Both models indicate β-lap treatment reroutes citrate out of the mitochondria to supply acetyl-CoA units. Differences in relative net flux values indicate a significant downregulation in glycolysis caused by β-lap as well as TCA cycle proper induced by both β-lap and AOA treatment in MDA-MB-231 *NQO1*^+^ cells. The relative flux ratios highlight significant decrements in glycolysis as well as the breakdown of the forward TCA cycle flux caused by β-lap action in NQO1^+^ cells. Abbreviation of metabolic reactions: lactate dehydrogenase (LDH), lactate export (Export), glyceraldehyde-3-phosohate dehydrogenase (GAPDH), pyruvate carboxylase (PC), pyruvate dehydrogenase (PDH), citrate synthase (CS), mitochondrial citrate carrier (CIC), citrate dehydrogenase (CDH), α-ketoglutarate dehydrogenase (AKGDH), succinate dehydrogenase (SDH), aspartate aminotransferase (AST), malate dehydrogenase (MDH), glutaminase (GLS), glutamate dehydrogenase (GDH), and fatty acid oxidation (FO). (Note: *n* = 3, biological replicate data are represented as mean ± SEM. Statistical significance was determined by ANOVA and student’s *t*-test post hoc analysis and is presented as: (**) if *p ≤* 0.01 and (***) if *p ≤* 0.001 compared to control. (α) if *p ≤* 0.05 and (γ) if *p ≤* 0.001 compared to β-lap treatment. (a) if *p ≤* 0.05 and (c) if *p ≤* 0.001 compared to AOA treatment). CoA, coenzyme A.

The large amount of data for fractional enrichment across the treatment groups demands metabolic modeling to more completely assimilate and understand the changes in metabolic flux caused by the treatments. By modeling the fractional enrichments and pool sizes, relative rates of flux through single enzymatic steps or reaction pathways can be computed. However, without an absolute rate as a reference, we were forced to develop two models that agree about flux through multiple pathways, but differ in detail (Supplementary Materials Figure S7). One model includes only glucose as a source of acetyl-CoA (glu-ox) while the other includes multiple sources of unlabeled acetyl-CoA (mult-ox). Both models assume the same arbitrary flux for glucose import and all subsequent reactions are relative to this flux. In Figure 8, MDA-MB-231 *NQO1*^+^ cells (Panel A and B) treated with β-lap and the combination strategy showed significantly lower glyceraldehyde-3-phosphate dehydrogenase (GAPDH) and lactate dehydrogenase (LDH) flux relative to glucose import. LDH-export denotes intracellular LDH net flux and lactate export (v7 and v8 in Supplementary Materials Figure S7). The α-ketoglutarate dehydrogenase-succinate dehydrogenase step (AKGDH-SDH) represents v14 and v15 (Supplementary Materials Figure S7), which must have equal flux as we do not include anaplerotic flux through succinate in the model. The models diverge significantly after the citrate dehydrogenase step, where the glu-ox model integrates lower fractional enrichments for compounds of the TCA cycle after citrate by significantly upregulating glutamine anaplerosis (Figure 8C, glutaminase (GLS) and glutamate dehydrogenase (GDH)). In contrast, the mult-ox model elevates the F0 fraction of unlabeled acetyl-CoA while simultaneously downregulating aspartate aminotransferase flux (Figure 8A). As these are relative rates, a selection of ratios for certain pathways was also calculated to emphasize changes in flux between the two models (Figure 8B,D). In the case of MDA-MB-231 *NQO1*^−^ cells (Supplementary Materials Figure S8), GAPDH and LDH fluxes across treatments were not significantly different. This minimal effect on glycolytic metabolism is in line with previous results for *NQO1*^−^ cells. [22]. From this analysis, one can observe that β-lap-treated MDA-MB-231 *NQO1*^+^ cells had significantly higher relative flux through pyruvate dehydrogenase (PDH), citrate synthase (CS), and mitochondrial citrate carrier (CIC) relative to glucose import, indicating that citrate is being exported out of the mitochondria. This rerouting of citrate leads to significant decrements in forward TCA cycle flux induced by treatment (Figure 8A). No other cell line showed a consistent decrease in forward flux. These data are in line with negligible changes in fractional enrichment observed between the treatment groups for NQO1^−^ cell lines from glutamate to malate suggesting no change in unlabeled substrate import and labeled substrate export between groups (Figure 7). Additionally, MDA-MB-231 *NQO1*^+^ cells showed increased malate dehydrogenase (MDH) flux in all treatments, including AOA alone, which was unlike other cell lines, which had mixed reactions to treatment (Supplementary Materials Figures S8–S10). It is notable that T47D cells showed minimal changes across fluxes (Supplementary Materials Figure S9) while MDA-MB-468 cells showed complex changes in all fluxes caused by treatment, which may be due to off-target effects in this cell line (Supplementary Materials Figure S10).

### 3.5. Coefficient of Drug Interaction Analysis

CDI values was calculated to determine the synergistic effect of AOA and β-lap combinatorial treatment on TCA cycle ^13^C enrichments. CDI values were determined for citrate, glutamate, and succinate m+2 isotopologues by analyzing fractional enrichment values. In Table 1, we report treatment/control (T/C) values for each treatment group used to calculate CDI, along with the metabolite-specific CDI value. Additionally, we included a column to show the observed effect of combinatorial treatment relative to control in the specific isotopologue distribution of metabolites. β-lap+AOA treatment in MDA-MB-231 *NQO1*^+^ cells demonstrates evidence for decreased label deposition in the TCA cycle caused by combinatorial treatment (Table 1). In contrast, CDI values are approximately equal to one in MDA-MB-231 *NQO1*^−^ cells, indicating a minor synergistic effect for citrate and succinate, and a slight antagonistic effect for glutamate. T47D and MDA-MB-468 exhibited minor effects based on CDI values (Supplementary Materials Table S1). 

## 4. Discussion

To combat the negative side effects of β-lap treatment, we proposed a novel combinatorial treatment that could potentially minimize β-lap toxicities while maintaining a high degree of therapeutic potency. In this study, we utilized β-lap and AOA to assess their anti-cancer effects against four different breast cancer cell lines, only one of which strongly expressed NQO1. We present the enhancement of β-lap action by demonstrating a synergistic downregulation of central metabolism caused by combinatorial treatment with AOA, but this effect did not result in an amplification of β-lap activity in causing cancer cell death (Supplementary Materials Figure S6). Nevertheless, complex multi-factorial effects on metabolic turnover were observed across the treatment groups.

NAD^+^-keresis is induced by a massive depletion of intracellular NAD^+^ caused by β-lap action [24]. One way to significantly offset and further drive this mechanism is to target other metabolic pathways that are highly dependent on NAD^+^/NADH. Our previous work has shown a significant reduction in flux through metabolic pathways that are vulnerable to NAD^+^/NADH imbalance including branched-chain amino acid metabolism and the malate-aspartate shuttle [23]. In this work, we targeted the malate-aspartate shuttle with AOA as a strategy to disconnect cytoplasmic and mitochondrial NADH pools in order to further disturb cellular redox balance and central metabolism. The uninterrupted bioenergetics of the cell requires a tightly controlled balance between NADH production and oxidation that effectively regulates electron transport [37]. The NAD^+^/NADH balance is maintained, in part, by the compartmentalization of cytoplasmic and mitochondrial NADH pools [38]. While NAD^+^ can be regenerated in the electron transport chain through mitochondrial NADH oxidation, cytoplasmic NADH can be generated by a variety of intermolecular shuttles [30]. The MAS is the major NADH shuttle system that moves electrons past the impermeable mitochondrial membrane by a series of NAD^+^/NADH redox transfer reactions [39]. AST and MDH mediate the MAS by cycling aspartate, malate, α-ketoglutarate, oxaloacetate, and glutamate through the cytoplasm and mitochondria [40]. In the cytoplasm, AST converts aspartate to the dicarboxylate oxaloacetate (impermeable to the mitochondrial membrane), which is then reduced to malate by MDH, which simultaneously oxidizes NADH [41]. Cytoplasmic malate can then be transported into the mitochondria by the malate-α-ketoglutarate antiporter [42]. Once in the mitochondria, MDH reduces NAD^+^ and converts malate to mitochondrial oxaloacetate that can serve as a substrate for citrate synthase, or is subsequently converted to aspartate, which can exit the mitochondria through the glutamate-aspartate antiporter reinitiating the cycle [42]. Several cancer cell lines and tumors contain a highly active malate-aspartate shuttle that has been estimated to contribute ~20% of the cellular respiratory rate, and depending on cell type, a significant component of NADH oxidation [31,42,43]. Therefore, we used AOA to inhibit the malate-aspartate shuttle during β-lap treatment.

We assessed treatment-induced metabolic perturbations by measuring changes in global metabolite pools and metabolic turnover from [U-^13^C]glucose. [U-^13^C]glucose tracer analysis has been well established and is frequently used as a primary technique in cancer metabolism research [43]. The [U-^13^C]glucose tracer is readily consumed by cancer cells, serves as a major component and precursor of several bioenergetic, biosynthetic, and redox pathways, and has been used to assess systemic changes with no measurable isotope effects on metabolic turnover and health of cells [43,44].

Using this technique, our results show a direct effect of drug action and a reduction in the levels of certain metabolites. Global metabolomic analysis (Figure 1) demonstrated treatment-dependent (Control, β-lap, AOA, and β-lap+AOA) separation in the intracellular pools of extracted metabolites, which were consistent with the NQO1 expression determined in this study. Western blot analysis showed that MDA-MB-231 *NQO1*^+^ cells express high levels of NQO1 protein while all three other cell lines were NQO1 deficient (Supplementary Materials Figure S5).

Analysis of ^13^C-lactate isotopologue enrichment indicates that β-lap action significantly hinders lactate secretion, leading to higher intracellular m+3 lactate labeling caused by glycolytic metabolism paired with diminished lactate efflux (Figure 2 and Figure 3, and Supplementary Materials Figure S2). While not fully understood, this phenotype has been consistently reported for cancer cells where NQO1 is targeted for bioactivation [22]. Interestingly, AOA treatment caused significantly higher intracellular m+3 lactate enrichment compared to control in NQO1^−^ cells (Figure 2C). Greater glycolytic activity in this case could be potentially compensating for diminished malate-aspartate shuttle activity. Moreover, AOA treatment caused a reduction in m+3 alanine labeling in all four cell lines, indicative of alanine aminotransferase inhibition as expected (Figure 4).

Comparative analysis of the citrate isotopologue distributions between MDA-MB-231 *NQO1*^+^ and MDA-MB-231 *NQO1*^−^ cells shows that AOA enhances β-lap drug action in downregulating TCA cycle enrichment (Figure 6). Lower citrate m+2 enrichment in combinatorial treatment suggests loss of NAD^+^ needed for pyruvate dehydrogenase flux in MDA-MB-231 *NQO1*^+^ cells. Citrate pool sizes were also dramatically reduced by β-lap treatment, which enforces strong constraints on the metabolic modeling (Supplementary Materials Figure S2). Significantly reduced succinate and glutamate m+2 labeling in NQO1^+^ cells further support that β-lap and AOA are synergistically downregulating the TCA cycle. Intracellular aspartate labeling in β-lap and AOA-treated MDA-MB-231 *NQO1*^+^ cells was significantly greater than the independent treatments, while the intracellular aspartate pool was significantly lower compared to β-lap and higher compared to AOA (Supplementary Materials Figures S2 and S3). This effect is likely due to AOA inhibition of AST1 (cytosolic AST) and AST2 (mitochondrial AST), which prevents aspartate to oxaloacetate conversion in the mitochondrial intermembrane space and causes a buildup of malate in the mitochondrial matrix [45]. In the combinatorial treatment, this effect is likely exacerbated by a decrease in MDH turnover due to β-lap-induced NAD^+^ depletion (Figure 7 and Supplementary Materials Figure S3). Interestingly, combinatorial treatment enhances β-lap action on aspartate labeling in an NQO1-dependent manner leaving the subject for further studies to characterize this effect. Analysis of TCA cycle intermediates m+3 enrichment showed a consistent trend in all treatments, indicating that some portion of TCA cycle labeling was likely provided by pyruvate carboxylase (PC).

From previous work, it is clearly understood that β-lap action downregulates metabolism through NAD^+^ and ATP depletion [17]. NAD^+^ loss should diminish the activity/flux of NAD^+^-dependent enzymes. To this end, we employed metabolic flux analysis by INCA and developed two models of central metabolic pathways and reactions such as glycolysis, TCA cycle, malate-aspartate shuttle, as well as relevant import and export pathways along with branching reactions (Supplementary Materials Figure S7 and Table S2). In doing so, we confirm changes in flux consistent with our predictions for β-lap treatments’ mode of action. We show decreases in flux through GAPDH of β-lap treated NQO1^+^ but not NQO1^−^ cells as well as LDH to lactate export relative to glucose import (based on intracellular and extracellular data). These changes correlate with decreases in glycolysis previously shown [22]. It is important to note that this observation is due to a decrement in lactate secretion [22]. This is evidenced by higher intracellular m+3 lactate enrichment in β-lap-treated NQO1^+^ cells, and lower extracellular m+3 lactate enrichment (Figure 2). The higher m+3 lactate enrichment in cells is likely caused by a buildup of lactate during the early time period of treatment, but as treatment progresses secretion is inhibited, trapping the accumulated m+3 lactate intracellularly. Surprisingly, while we observed significant decrements in glycolytic flux, PDH flux was significantly upregulated in β-lap-treated NQO1^+^ cells relative to glucose import in both models (Figure 8). This increase in PDH flux is matched by nearly identical increases in CS and CIC flux, which indicate citrate production with subsequent export out of the mitochondria. As the quantity of citrate is depleted globally in the β-lap-treated NQO1^+^ cells, we hypothesize the exported citrate may be broken down into acetyl-CoA and oxaloacetate subunits by ATP citrate lyase for DNA repair pathways [46]. As the efflux of citrate is matched by an influx of malate mitochondrially, which contributes to greater availability of oxaloacetate subunits for CS flux, a minimal metabolic cycle bypasses the majority of the NAD^+^ dependent enzymes in the mitochondria, acting to sustain minimal flux needed for overall survival [47]. Elevated CIC flux in the β-lap-treated cells relative to control also leads to a depletion of substrate from the mitochondrial TCA cycle intermediates such as citrate and α-ketoglutarate, which appears to extend to succinate (Figure 8 and Supplementary Materials Figure S2).

While the first presented model, mult-ox, (Figure 8A,B) clearly addresses most of the expected mechanisms of β-lap action, it has a limited explanation for the dilution of glutamate fractional enrichment versus that of citrate (Figure 6 and Figure 7). Based on decreases in glutamate m+2 enrichment and glutamate pools caused by β-lap treatment, we suspected that the depletion in glutamate label would be caused by increased GLS and glutamate anaplerosis as a way to attenuate TCA cycle decrements [29]. This effect was not observed in the mult-ox model (Figure 8A,B and Supplementary Materials Figure S7). To address this discrepancy, we developed an alternative model (Figure 8 and Supplementary Materials Figure S7) by excluding the contributions of unlabeled acetyl-CoA derived from fatty acid import and oxidation, glu-ox. The alternative model shows that halting NAD^+^ metabolism in NQO1^+^ cells by β-lap led to an increased proportion of α-ketoglutarate derived from glutamate rather than the oxidation of citrate (Figure 8C,D). This claim is backed by a depletion of glutamate in β-lap-treated NQO1^+^ cells, but not NQO1^−^ cells (Figure 7 and Supplementary Materials Figure S2). TCA cycle proper also appears disrupted as the amount of succinate is significantly decreased by the addition of β-lap (Supplementary Materials Figure S2). AKGDH-SDH flux relative to CDH and glutamate anaplerosis highlights decreases in the turnover of the TCA cycle proper (Figure 8C,D). At the 2 h post-treatment time point, some metabolic pathways dependent on NAD^+^ (poly-ADP ribose polymerase-1) may still be active in DNA repair. Our models propose that the dilution of the citrate labeling from ~40% or higher to ~8% or lower enrichment in glutamate resulted from increased glutamine import relative to the labeled glucose utilization to make acetyl-CoA. These data collectively support the notion that in the case of β-lap treatment, total glycolytic flux is down, while other substrates in the media such as glutamine play a larger role in supplying anaplerotic substrates for TCA cycle function. We have also been able to use this information to show potential nuances of β-lap treatment and show that AOA treatment concomitantly decreased AST and AKGDH-SDH flux in NQO1^+^ cells.

## 5. Conclusions

Our experimental design was sound, but the loss of media samples due to incorrect storage did not allow us to estimate glucose consumption. This quantitative rate could have been used to reference our relative rates from the two proposed models, and more than likely would have allowed us to identify a single model that explained the data in detail. Without this constraint, we cannot definitively state that β-lap treatment causes increased glutamine anaplerosis. Future experiments will directly address this question.

In conclusion, we demonstrated that β-lap and AOA combinatorial treatment can synergistically reduce the turnover of central metabolism and that [U-^13^C]glucose tracer analysis can be used to identify anti-cancer metabolic synergies. While AOA was not synergistic for causing cell death in this case, this does not exclude the possibility that interruption of other shuttles, such as the glycerol-3-phosphate shuttle, might show increased efficacy. The significant effects on central metabolism indicate that metabolic imaging using [^2^H_7_]glucose might potentially be used to monitor anti-tumorigenic effects in vivo and demonstrate similar results observed in this study [48].

## Figures and Tables

**Table 1:** Synergistic effects in MDA-MB-231 *NQO1*^+^ and *NQO1*^−^ breast cancer cells determined by CDI values.

Cell Line	Metabolite	Isotopologue Fractional Enrichment (T/C)			CDI	Effect
		β-Lap	AOA	β-Lap+AOA		
NQO1^+^	Citrate	0.94 ± 0.01	0.98 ± 0.01	0.86 ± 0.01	0.93 ± 0.04	Decrease
	Glutamate	0.60 ± 0.00	1.03 ± 0.01	0.50 ± 0.01	0.80 ± 0.12	Decrease
	Succinate	0.58 ± 0.00	0.96 ± 0.00	0.35 ± 0.01	0.64 ± 0.18	Decrease
NQO1^−^	Citrate	1.05 ± 0.00	1.04 ± 0.01	1.04 ± 0.00	0.96 ± 0.01	Increase
	Glutamate	1.04 ± 0.00	1.02 ± 0.00	1.14 ± 0.00	1.07 ± 0.01	Increase
	Succinate	1.11 ± 0.00	1.08 ± 0.00	1.17 ± 0.00	0.97 ± 0.02	Increase

Coefficient of drug interaction (CDI) values demonstrates AOA and β-lap NQO1 selective synergy in the downregulation of the TCA cycle in MDA-MB-231 cells. (Note: citrate, glutamate, and succinate m+2 isotopologue data are represented as fractional enrichment (T/C) ± SEM). CDI, coefficient of drug interaction; β-lap, β-lapachone; AOA, aminooxyacetic acid; NQO1, quinone oxidoreductase 1; TCA, tricarboxylic acid; T/C, treatment/control; SEM, standard error of the mean.

## Data Availability

The data presented are available in this research article and in the Supplementary Materials section and can also be obtained from the corresponding author upon reasonable request.

## Supplementary Materials

The following are available online at https://www.mdpi.com/article/10.3390/nu14153020/s1, Figure S1: Schematic representation of the experiment timeline, Figure S2: Normalized intensity of intracellular metabolite pools, Figure S3: Intracellular aspartate isotopologue distribution, Figure S4: Analysis of TCA cycle activity in T47D and MDA-MB-468 breast cancer cells, Figure S5: NQO1 protein expression levels, Figure S6: Cell viability analysis in MDA-MB-231 *NQO1*^+^ and *NQO1*^−^ cells, Figure S7: Representative diagram of the flux model analysis, Figure S8: Flux model analysis in MDA-MB-231 *NQO1*^−^, Figure S9: Flux model analysis in T47D, Figure S10: Flux model analysis in MDA-MB-468, Table S1: Synergistic effects in T47D and MDA-MB-468 breast cancer cells determined by CDI values, Table S2: Metabolic flux model reactions.
