# Supplementary material for: Synergistic Effect of β-Lapachone and Aminooxyacetic Acid on Central Metabolism in Breast Cancer
Source: Nutrients. 2022 Jul 22;14(15):3020. doi: 10.3390/nu14153020 (PMC9331106; doi:10.3390/nu14153020)
Supplement: Supplementary file 1 [file nutrients-14-03020-s001.zip › nutrients-1820323-supplementary.pdf]

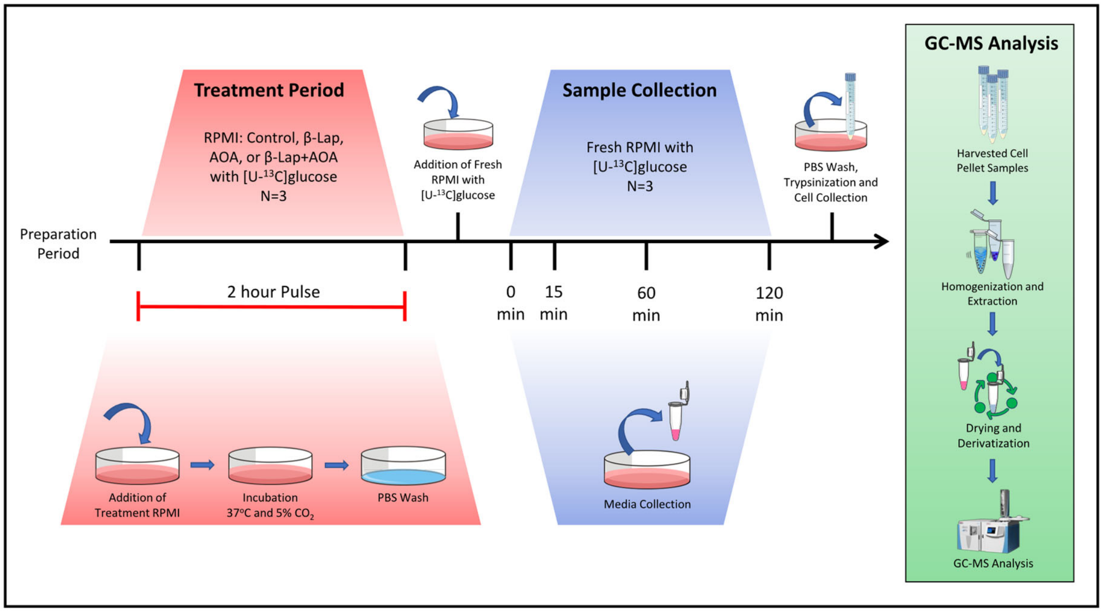

Figure S1. Schematic representation of the experiment timeline. Prior to initiating the experiment, all reagents and media were prepared during the preparation period. The experiment began upon the addition of treatment RPMI (Control, β-lap, AOA, or β-lap+AOA) containing [U-^{13}C]glucose tracer. Cells were incubated in treatment media for 2 h and then immediately washed with PBS and incubated with fresh RPMI with [U-^{13}C]glucose for an additional 2 h. During the final 2 h incubation, media sample points were collected. At the end of the experiment, cells were washed with PBS, harvested by trypsinization, and processed for GC-MS analysis. Sample preparation for GC-MS analysis involved homogenizing and extracting cell pellets, drying media samples and cell extracts, derivatizing all dried samples, and loading samples into GC vials. RPMI, Roswell Park Memorial Institute Medium; β-lap, β-lapachone; AOA, aminooxyacetic acid; PBS, phosphate buffered saline; GC-MS, gas chromatography–mass spectrometry.

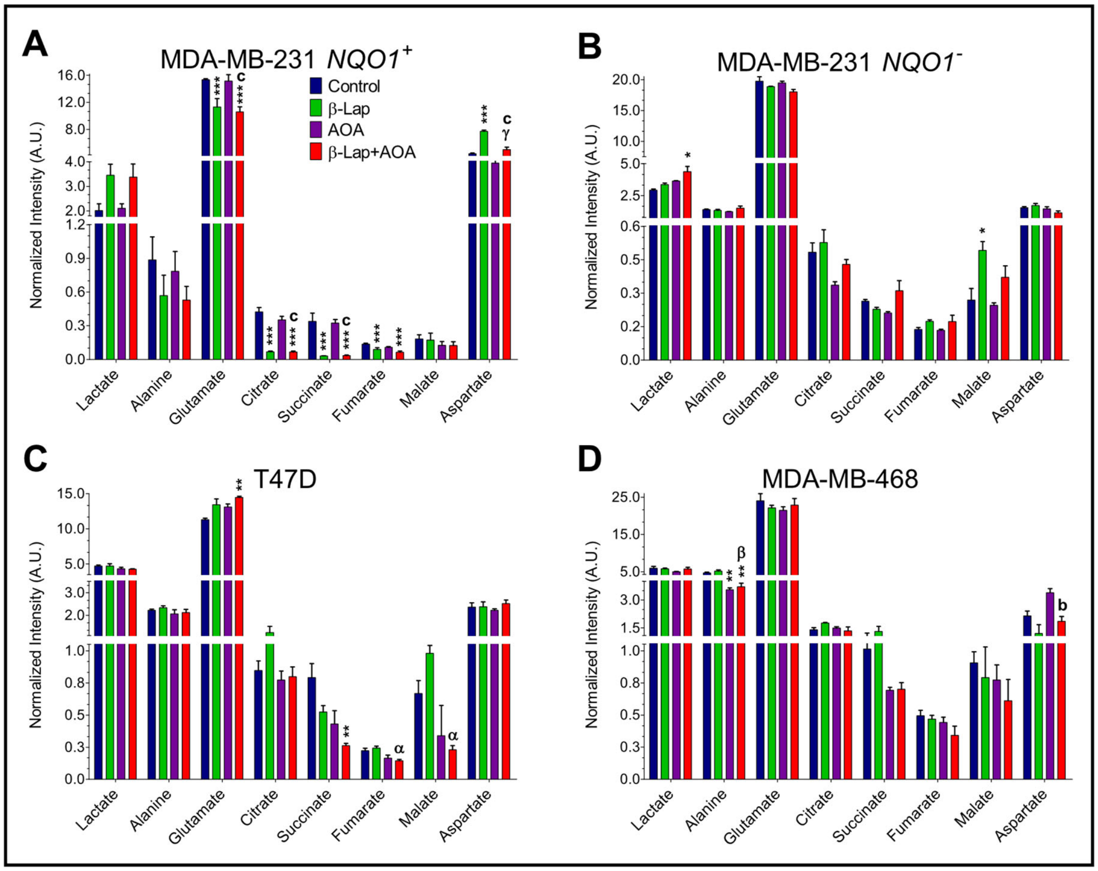

Figure S2. Normalized intensity of intracellular metabolite pools in MDA-MB-231 *NQO1*⁺ (**A**), MDA-MB-231 *NQO1*⁻ (**B**), T47D (**C**), and MDA-MB-468 (**D**) breast cancer cells. (Note: $n = 3$, biological replicate data is represented as mean ± SEM. Statistical significance was determined by ANOVA and student's t-test post hoc analysis and is presented as: (*) if $p \leq 0.05$, (**) if $p \leq 0.01$, and (***) if $p \leq 0.001$ compared to control. (α) if $p \leq 0.05$, (β) if $p \leq 0.01$, and (γ) if $p \leq 0.001$ compared to β-lap treatment. (b) if $p \leq 0.01$ and (c) if $p \leq 0.001$ compared to AOA treatment.). NQO1, quinone oxidoreductase 1; SEM, standard error of the mean; A.U., arbitrary units.

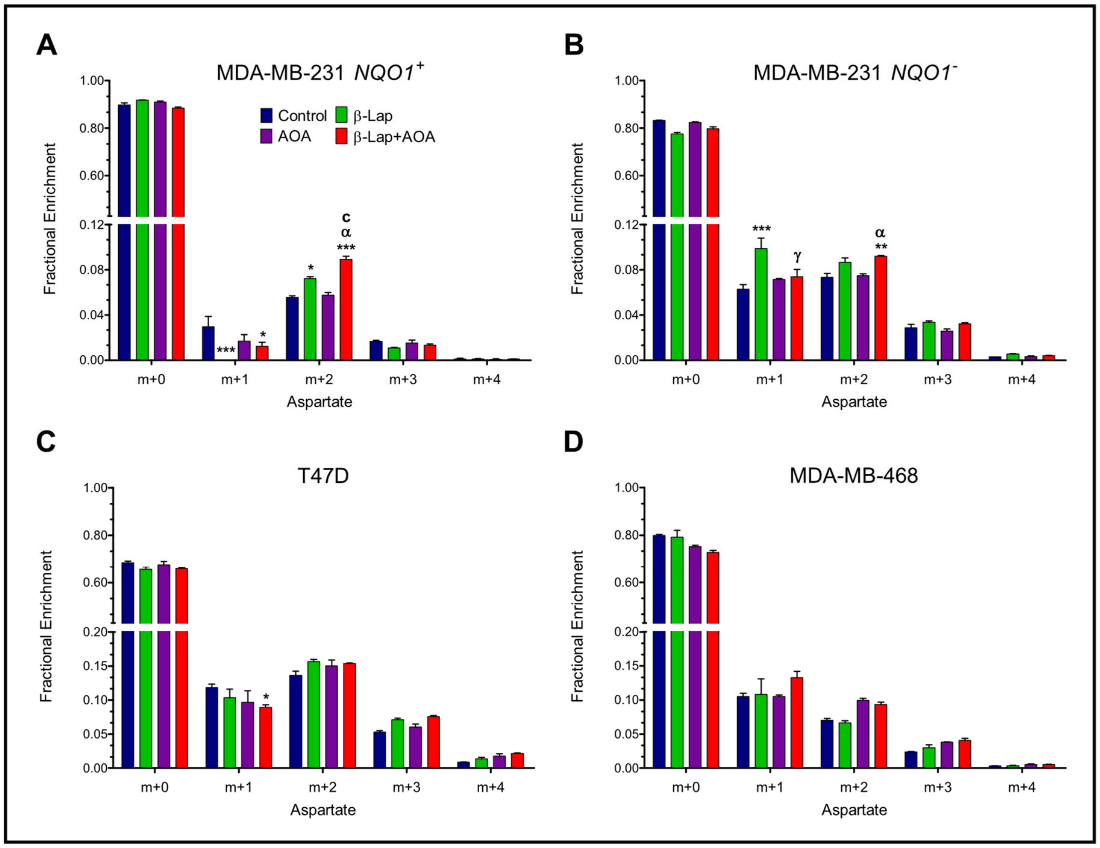

Figure S3. Intracellular aspartate isotopologue distribution in MDA-MB-231 *NQO1*⁺ (**A**), MDA-MB-231 *NQO1*⁻ (**B**), T47D (**C**), and MDA-MB-468 (**D**) breast cancer cells. (Note: $n = 3$, biological replicate data is represented as mean ± SEM. Statistical significance was determined by ANOVA and student's t-test post hoc analysis and is presented as: (*) if $p \leq 0.05$, (**) if $p \leq 0.01$, and (***) if $p \leq 0.001$ compared to control. (α) if $p \leq 0.05$ and (γ) if $p \leq 0.001$ compared to β-lap treatment. (c) if $p \leq 0.001$ compared to AOA treatment.).

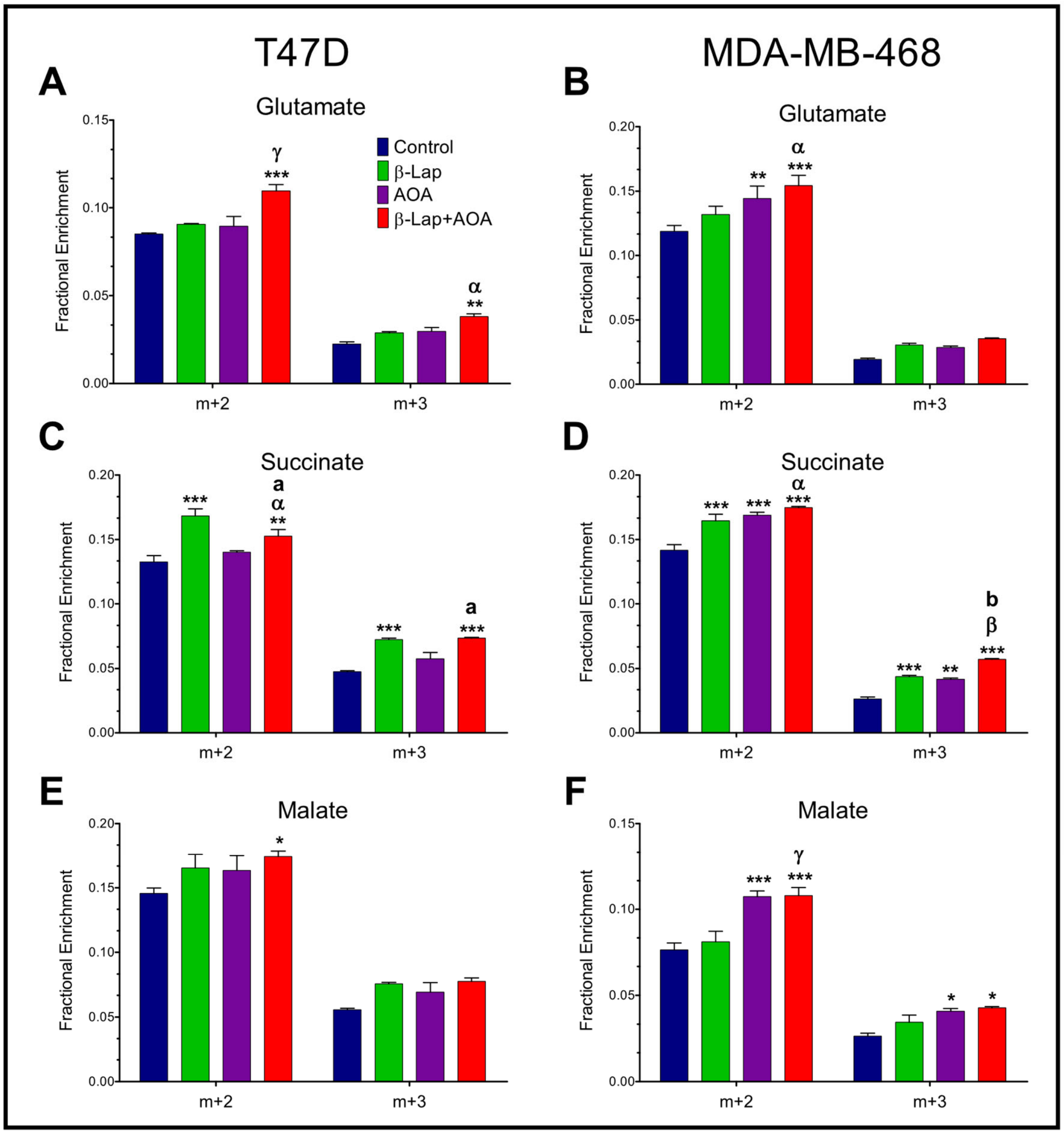

Figure S4. Analysis of TCA cycle metabolite enrichments in T47D (**A**,**C**,**E**) and MDA-MB-468 (**B**,**D**,**F**) breast cancer cells. (Note: $n = 3$, biological replicate data is represented as mean ± SEM. Statistical significance was determined by ANOVA and student's t-test post hoc analysis and is presented as: (*) if $p \leq 0.05$, (**) if $p \leq 0.01$, and (***) if $p \leq 0.001$ compared to control. (α) if $p \leq 0.05$, (β) if $p \leq 0.01$, and (γ) if $p \leq 0.001$ compared to β-lap treatment. (a) if $p \leq 0.05$ and (b) if $p \leq 0.01$ compared to AOA treatment). TCA, tricarboxylic acid.

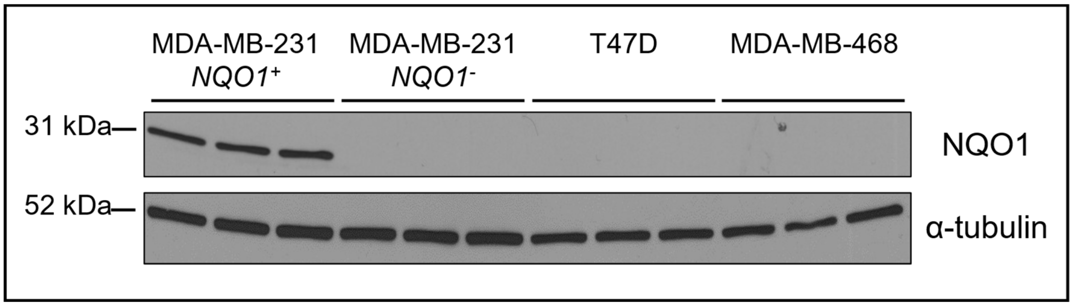

Figure S5. NQO1 protein expression levels of MDA-MB-231 *NQO1⁺*, MDA-MB-231 *NQO1⁻*, T47D, and MDA-MB-468 breast cancer cells. Western blot was performed on n = 3 biological replicates.

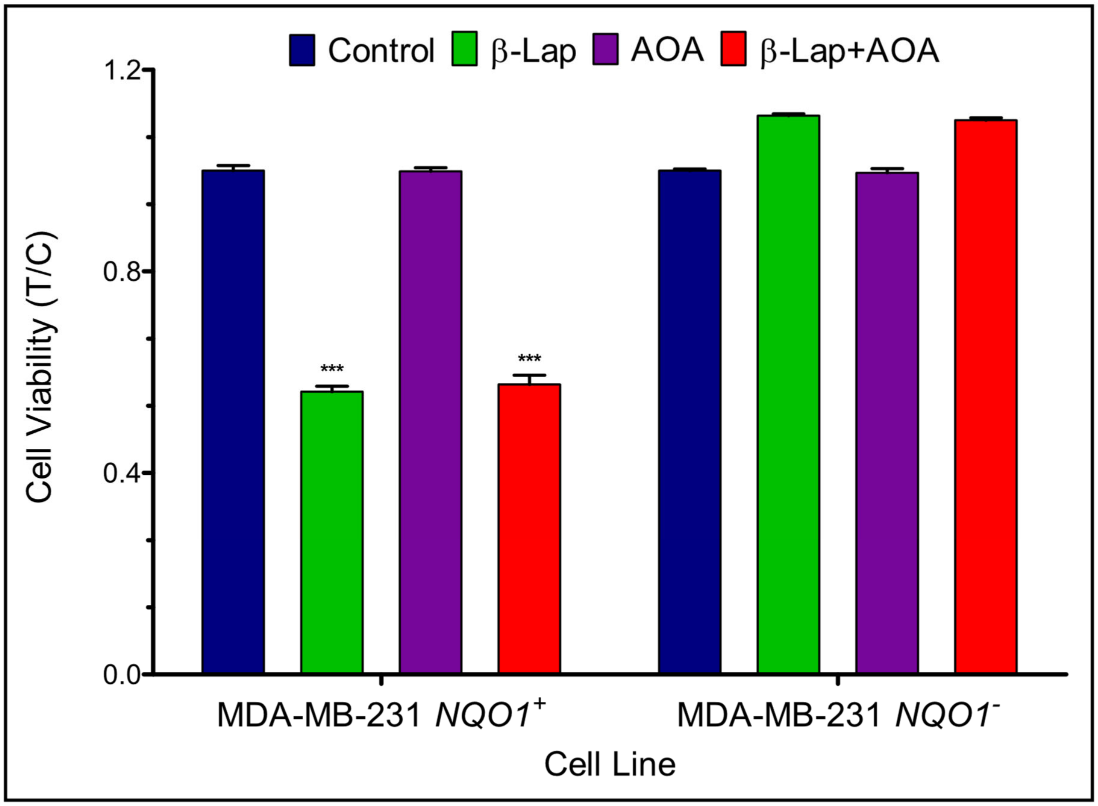

Figure S6. Cell viability analysis in MDA-MB-231 *NQO1*+ and *NQO1*− triple negative breast cancer cells. An MTT assay was performed on $n = 3$ biological replicates. Significantly lower viability was observed in NQO1+ cells treated with β-lap and combinatorial treatments, while no significance was observed in NQO1− cells across treatments. Statistical significance was determined by student's t-test analysis and is presented as: (***) if $p \leq 0.001$ compared to control. MTT, 3-[4,5-dimethylthiazol-2-yl]-2,5 diphenyl tetrazolium bromide.

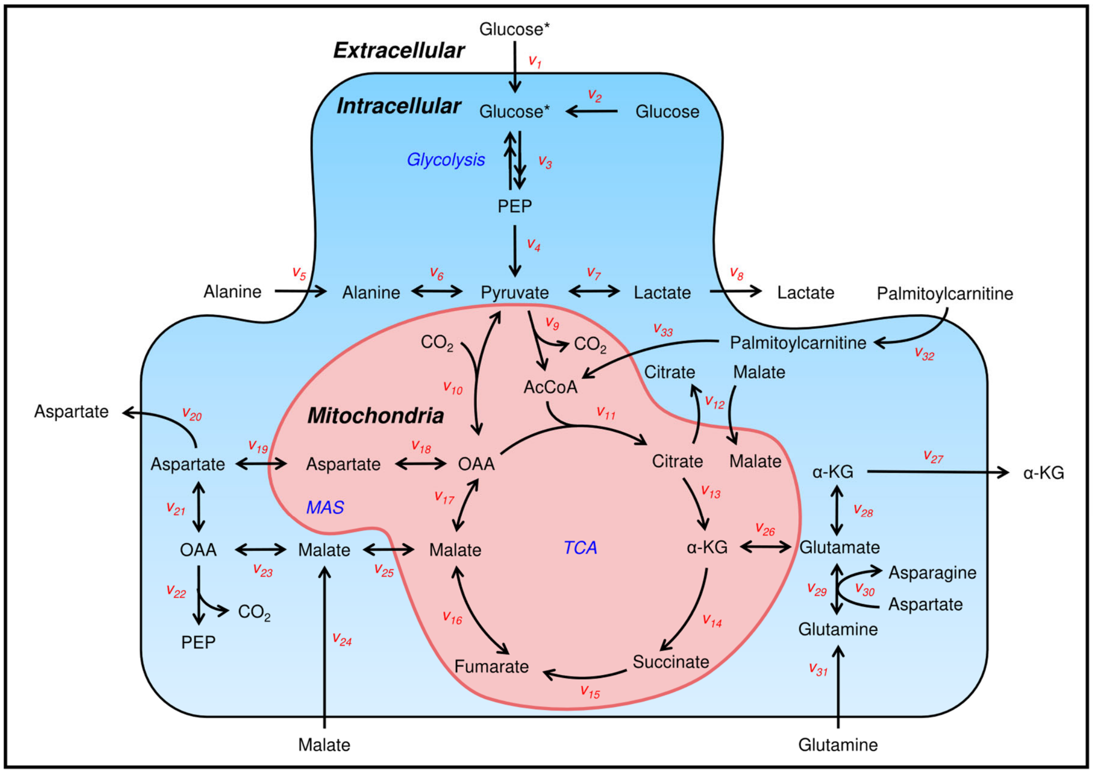

Figure S7. Representative diagram of the flux model analysis. The glu-ox model suppresses the v32 and v33 flux from palmitate, while mult-ox allows for sources of acetyl-CoA from other substrates in the media. Metabolites are represented in black text and flux reactions are in red. Labeled glucose is denoted by a (*). Specific flux reactions are further detailed in Supplementary Materials Table S2. CoA, coenzyme A; PEP, phosphoenolpyruvate; AcCoA, acetyl-coenzyme A; OAA, oxaloacetic acid; α-KG, alpha ketoglutarate, MAS, malate-aspartate shuttle.

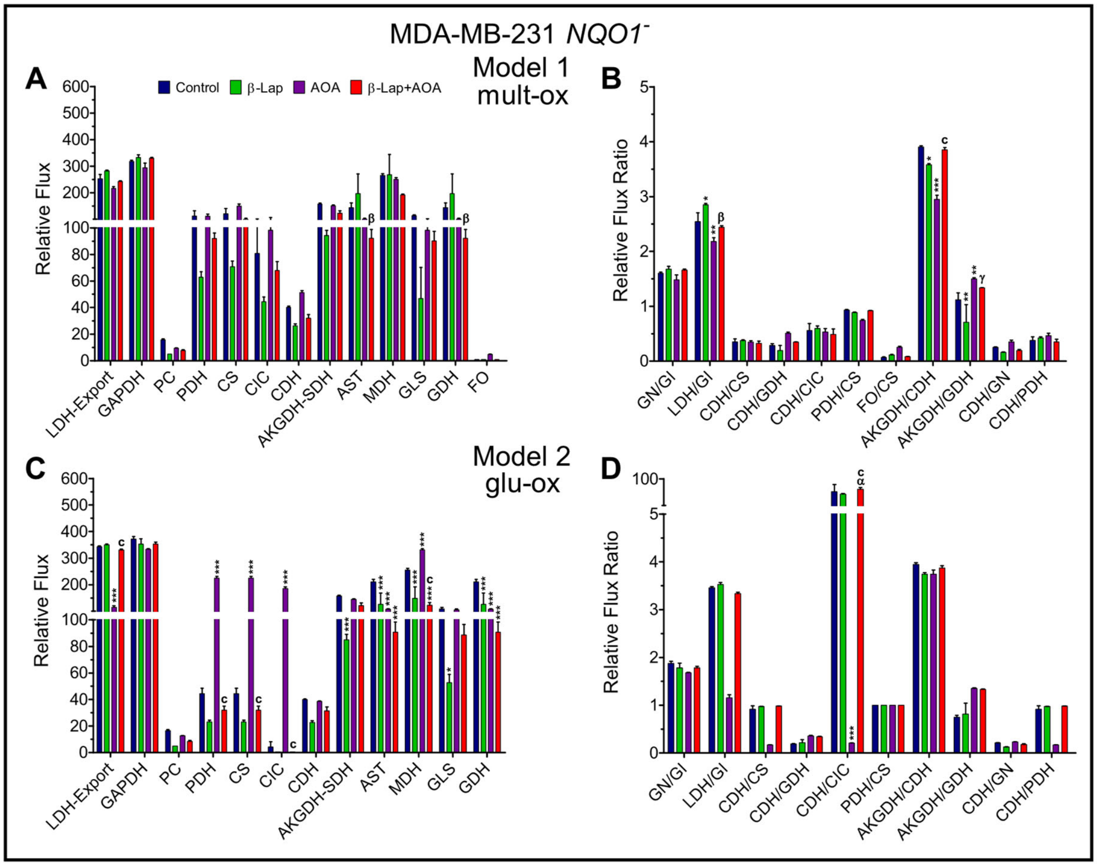

Figure S8. Flux model analysis in MDA-MB-231 *NQO1*⁻. Model 1 is represented in relative fluxes and relative flux ratios in panels (**A**,**B**), and Model 2 is represented in panels (**C**,**D**). Abbreviation of metabolic reactions: lactate dehydrogenase (LDH), lactate export (Export), glyceraldehyde-3-phosohate dehydrogenase (GAPDH), pyruvate carboxylase (PC), pyruvate dehydrogenase (PDH), citrate synthase (CS), mitochondrial citrate carrier (CIC), citrate dehydrogenase (CDH), α-ketoglutarate dehydrogenase (AKGDH), succinate dehydrogenase (SDH), aspartate aminotransferase (AST), malate dehydrogenase (MDH), glutaminase (GLS), glutamate dehydrogenase (GDH), and fatty acid oxidation (FO). (Note: $n = 3$, biological replicate data is represented as mean ± SEM. Statistical significance was determined by ANOVA and student's t-test post hoc analysis and is presented as: (*) if $p \leq 0.05$, (**) if $p \leq 0.01$, and (***) if $p \leq 0.001$ compared to control. (α) if $p \leq 0.05$, (β) if $p \leq 0.01$, and (γ) if $p \leq 0.001$ compared to β-lap treatment. (c) if $p \leq 0.001$ compared to AOA treatment.).

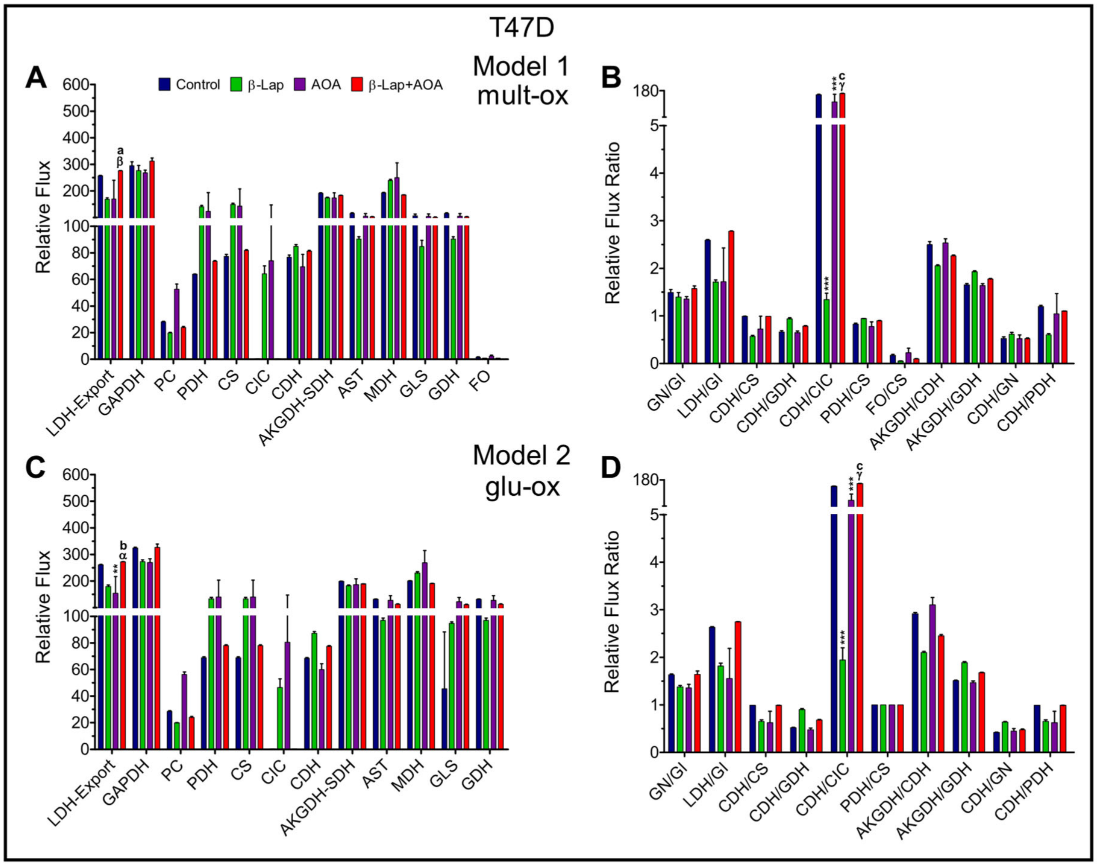

Figure S9. Flux model analysis in T47D. Model 1 is represented in relative fluxes and relative flux ratios in panels (**A**,**B**), and Model 2 is represented in panels (**C**,**D**). Abbreviation of metabolic reactions: lactate dehydrogenase (LDH), lactate export (Export), glyceraldehyde-3-phosohate dehydrogenase (GAPDH), pyruvate carboxylase (PC), pyruvate dehydrogenase (PDH), citrate synthase (CS), mitochondrial citrate carrier (CIC), citrate dehydrogenase (CDH), α-ketoglutarate dehydrogenase (AKGDH), succinate dehydrogenase (SDH), aspartate aminotransferase (AST), malate dehydrogenase (MDH), glutaminase (GLS), glutamate dehydrogenase (GDH), and fatty acid oxidation (FO). (Note: $n = 3$, biological replicate data is represented as mean ± SEM. Statistical significance was determined by ANOVA and student's t-test post hoc analysis and is presented as: (**) if $p \leq 0.01$ and (***) if $p \leq 0.001$ compared to control. (α) if $p \leq 0.05$, (β) if $p \leq 0.01$, and (γ) if $p \leq 0.001$ compared to β-lap treatment. (a) if $p \leq 0.05$, (b) if $p \leq 0.01$, and (c) if $p \leq 0.001$ compared to AOA treatment.).

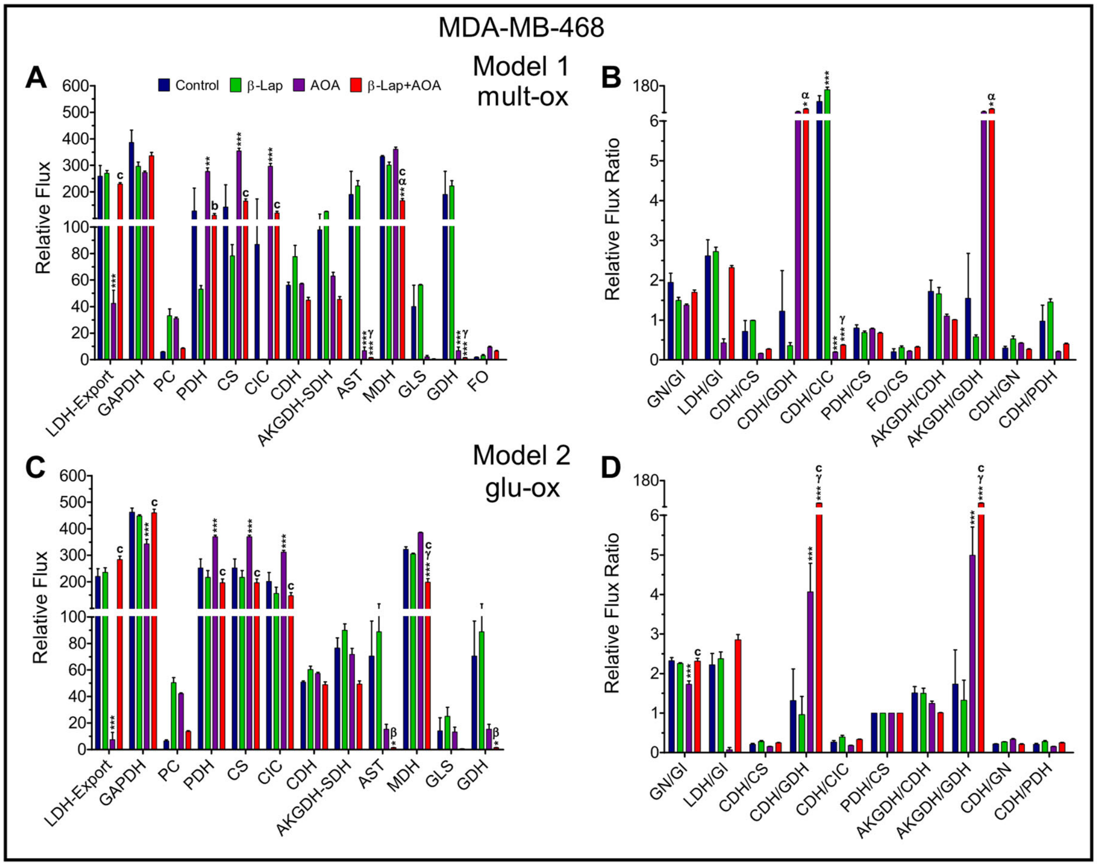

Figure S10. Flux model analysis in MDA-MB-468. Model 1 is represented in relative fluxes and relative flux ratios in panels (**A**,**B**), and Model 2 is represented in panels (**C**,**D**). Abbreviation of metabolic reactions: lactate dehydrogenase (LDH), lactate export (Export), glyceraldehyde-3-phosohate dehydrogenase (GAPDH), pyruvate carboxylase (PC), pyruvate dehydrogenase (PDH), citrate synthase (CS), mitochondrial citrate carrier (CIC), citrate dehydrogenase (CDH), α-ketoglutarate dehydrogenase (AKGDH), succinate dehydrogenase (SDH), aspartate aminotransferase (AST), malate dehydrogenase (MDH), glutaminase (GLS), glutamate dehydrogenase (GDH), and fatty acid oxidation (FO). (Note: N=3, biological replicate data is represented as mean ± SEM. Statistical significance was determined by ANOVA and student's t-test post hoc analysis and is presented as: (*) if $p \leq 0.05$, (**) if $p \leq 0.01$, and (***) if $p \leq 0.001$ compared to control. (α) if $p \leq 0.05$, (β) if $p \leq 0.01$, and (γ) if $p \leq 0.001$ compared to β-lap treatment. (b) if $p \leq 0.01$ and (c) if $p \leq 0.001$ compared to AOA treatment.).

Table S1. Synergistic effects in T47D and MDA-MB-468 breast cancer cells determined by CDI values.

Cell Line	Metabolite	Isotopologue Fractional Enrichment (T/C)			CDI	Effect
		**β-Lap**	**AOA**	**AOA+β-Lap**		
T47D	Citrate	0.97 ± 0.02	0.95 ± 0.01	0.93 ± 0.01	1.02 ± 0.04	Decrease
	Glutamate	1.07 ± 0.00	1.05 ± 0.01	1.29 ± 0.00	1.15 ± 0.04	Increase
	Succinate	1.27 ± 0.01	1.06 ± 0.00	1.15 ± 0.01	0.86 ± 0.03	Increase
MDA-MB-468	Citrate	1.02 ± 0.00	1.03 ± 0.00	0.95 ± 0.00	0.91 ± 0.00	Decrease
	Glutamate	1.11 ± 0.01	1.21 ± 0.01	1.30 ± 0.01	0.96 ± 0.05	Increase
	Succinate	1.16 ± 0.01	1.19 ± 0.00	1.23 ± 0.00	0.89 ± 0.00	Increase

Coefficient of drug interaction (CDI) values assessing AOA and β-lap synergy in the downregulation of the TCA cycle in T47D and MDA-MB-468 cells. (Note: citrate, glutamate, and succinate m+2 isotopologue data are represented as fractional enrichment (T/C) ± SEM). β-lap, β-lapachone; AOA, aminooxyacetic acid; TCA, tricarboxylic acid; SEM, standard error of the mean; T/C, treatment/control.

Table S2. Metabolic flux model reactions.

Reaction	Equation
(v1) Glucose Import	Glucose.media -> Glucose.cell
(v2) Unlabeled Glucose Contribution	Glucose -> Glucose.cell
(v3) Glycolysis Net	Glucose.cell <-> PEP + PEP
(v4) Pyruvate Kinase	PEP -> Pyruvate
(v5) Alanine Media	Alanine.media -> Alanine
(v6) Alanine Amino Transferase Net	Pyruvate <-> Alanine
(v7) Lactate Dehydrogenase Net	Pyruvate + 0.5 × O2 <-> Lactate
(v8) Lactate Export	Lactate -> Lactate.media
(v9) Pyruvate Dehydrogenase	Pyruvate + 0.5 × O2 -> AcCoA + CO2
(v10) Pyruvate Carboxylase	Pyruvate + CO2 -> OAA
(v11) Citrate Synthase	AcCoA + OAA -> Citrate
(v12) Mitochondrial Citrate Carrier	Citrate + Malate.cell -> Citrate.cell + Malate
(v13) Citrate Dehydrogenase	Citrate + 0.5 × O2 -> a_ketoglutarate + CO2
(v14) Alpha-Ketoglutarate Dehydrogenase	a_ketoglutarate + 0.5 × O2 -> Succinate + CO2
(v15) Succinate Dehydrogenase	Succinate + 0.5 × O2 <-> Fumarate
(v16) Fumarase	Fumarate <-> Malate
(v17) Malate Dehydrogenase	Malate + 0.5 × O2 <-> OAA
(v18) Aspartate Aminotransferase	OAA <-> Aspartate.mito
(v19) Glutamate-Aspartate Antiporter	Aspartate.mito + Glutamate -> Aspartate + Glutamate.mito
(v20) Aspartate Export	Aspartate -> Aspartate.out
(v21) Aspartate Aminotransferase	Aspartate -> OAA.cell
(v22) Phosphoenolpyruvate Carboxykinase	OAA -> PEP + CO2
(v23) Malate Dehydrogenase	OAA.cell -> Malate.cell
(v24) Malate Media Import	Malate.media -> Malate.cell
(v25) Malate Mitochondrial Import	Malate.cell + a_ketoglutarate -> Malate + a_ketoglutarate.cell
(v26) Glutamate Dehydrogenase	Glutamate.mito <-> a_ketoglutarate
(v27) Alpha-Ketoglutarate Export	a_ketoglutarate.cell -> a_ketoglutarate.out
(v28) Glutamate Dehydrogenase	a_ketoglutarate.cell -> Glutamate
(v29) Glutaminase Net	Glutamate <-> Glutamine
(v30) Asparagine Synthetase	Aspartate + Glutamine -> Asparagine + Glutamate
(v31) Glutamine Import	Glutamine.media -> Glutamine
(v32) Fatty Acid Import	Palmitoylcarnitine.media -> Palmitoylcarnitine
(v33) Fatty Acid Oxidation	Palmitoylcarnitine + 7 × O2 -> 8 × AcCoA
CO2_in	CO2.in -> CO2
CO2_out	CO2 -> CO2.out
O2_in	O2.in -> O2
O2_out	O2 -> O2.out

CO2, carbon dioxide; O2, oxygen; PEP, phosphoenolpyruvate; AcCoA, acetyl-coenzyme A; OAA, oxaloacetic acid.
